# The Effect of Liraglutide on the Hypolipidemic, Anti-Inflammatory, and Antioxidant Properties of Atorvastatin Mediated via the Nrf2/HO-1 Signaling Pathway: In Vivo and In Silico Validation

**DOI:** 10.3390/pharmaceutics18040490

**Published:** 2026-04-16

**Authors:** Sherif A. Kamar, Yosra M. Magdy, Tamer M. M. Abuamara, Amina A. Sedky, Tahani Mohamed Ibrahim Al-Hazani, Maha Alhelf, Eman Serry Zayed, Tarek A. Yousef, Abdullah Al-Dakhil, Mortaga M. Abou-Krisha, Samah J. Almehmadi, Sara Khedr

**Affiliations:** 1 Department of Basic Medical Science, Faculty of Dentistry, Al-Ahliyya Amman University, Amman P.O. Box 19111, Jordan; s.qamar@ammanu.edu.jo (S.A.K.); t.abuamara@ammanu.edu.jo (T.M.M.A.); 2 Department of Anatomy and Embryology, Faculty of Medicine, Ain Shams University, Cairo 11566, Egypt; 3 Department of Clinical Pharmacology, Faculty of Medicine, Ain Shams University, Cairo 11566, Egypt; y_yosra@outlook.com (Y.M.M.); aminasedky@med.asu.edu.eg (A.A.S.); sara_adel08@yahoo.com (S.K.); 4 Department of Histology, Faculty of Medicine, Al-Azhar University, Cairo 11651, Egypt; 5 Biology Department, College of Science and Humanities, Prince Sattam Bin Abdulaziz University, Al-Kharj 16273, Saudi Arabia; t.alhazani@psau.edu.sa; 6 Biotechnology School, Nile University, Giza 12677, Egypt; drmahasalah@kasralainy.edu.eg; 7 Medical Biochemistry and Molecular Biology Department, Faculty of Medicine, Cairo University, Cairo 12613, Egypt; 8 Department of Clinical Biochemistry, Faculty of Medicine, University of Tabuk, Tabuk 71491, Saudi Arabia; ezayed@ut.edu.sa; 9 Chemistry Department, College of Science, Imam Mohammad Ibn Saud Islamic University (IMSIU), Riyadh 11623, Saudi Arabia; mmaboukrisha@imamu.edu.sa; 10 Department of Clinical Laboratory Sciences, Faculty of Applied Medical Sciences, Umm Al-Qura University, Makkah, Saudi Arabia; sjamehmadi@uqu.edu.sa

**Keywords:** obesity, liraglutide, Nrf2/HO-1, atorvastatin, hyperlipidemia, inflammation

## Abstract

**Introduction**: Oxidative stress and inflammation are major factors linked to obesity and metabolic dysfunction, leading to a significantly higher risk of related diseases. Atorvastatin and liraglutide possess lipid-lowering, antioxidant, and anti-inflammatory effects that could synergistically improve obesity-related perturbations through modulation of the Nrf2/HO-1 signaling pathway. **Methodology**: We assessed liraglutide’s pharmacological potential in extending atorvastatin’s benefit on obesity, hyperlipidemia, and fatty liver in rats fed a high-fat diet (HFD) for 12 weeks. We specifically evaluated the effects of liraglutide treatment on atorvastatin-induced anti-inflammatory and antioxidant mechanisms, with a particular focus on Nrf2/HO 1 modulation in adipose and hepatic tissue. In silico analyses, including molecular docking and AlphaFold- Multimer modeling, evaluated the binding affinities of atorvastatin and liraglutide to Nrf2 and HO 1. **Results**: Compared to ND, the HFD-fed rats had a significantly higher final body weight (362.4 ± 12.7 g vs. 245.6 ± 9.8 g in ND, *p* < 0.05). There was a marked increase in serum total cholesterol (178.6 ± 9.2 mg/dL vs. 98.3 ± 6.4), fasting glucose (340.1 ± 8.2 mg/dL vs. 82.3 ± 3.1), HbA1c (7.8 ± 0.3 vs. 4.5 ± 0.2), and hepatic COX-2 expression (99.9 ± 6.3 vs 19.6 ± 2.4). The oxidative stress markers were also disturbed, as indicated by SOD (42.5 ± 3.1 vs. 95.2 ± 4.6 U/mg protein), GSH (18.3 ± 1.5 vs. 42.7 ± 2.8 nmol/mg), and p62 (0.005 ± 0.001 vs. 0.125 ± 0.01). Atorvastatin lowered cholesterol (121.2 ± 7.5 mg/dL), COX-2 (61.3 ± 3.3), and body weight (301.7 ± 11.5 g) compared to HFD. Meanwhile, liraglutide caused a greater reduction in body weight (268.5 ± 10.3 g), glucose (112.5 ± 6.7 mg/dL), and COX-2 (42.2 ± 2.9) than atorvastatin. The combination therapy produced the most significant effects, returning body weight (253.6 ± 9.1 g) to baseline, normalizing glucose and lipids, reducing COX-2 to 22.9 ± 2.0, and reactivating the Nrf2/HO-1 pathway, as shown by increased HO-1 expression and the restoration of p62 levels (0.078 ± 0.004). In silico analyses suggest that atorvastatin favorably binds to Nrf2 and HO-1, while liraglutide interacts with structurally relevant interfaces on these proteins, providing a mechanistic basis for their complementary antioxidant and cytoprotective effects. **Conclusions**: Our findings support targeting the Nrf2/HO-1 signaling pathway as a potential therapy for reversing hyperlipidemia and preventing mediators of inflammation and oxidative stress damage in the liver tissue. The evidence of increased efficacy observed with the combined atorvastatin and liraglutide supports a potential novel understanding of the complementary effects of atorvastatin and liraglutide. This finding requires further investigation to elucidate the combination’s therapeutic advantages in treating metabolic disorder scenarios.

## 1. Introduction

Metabolic diseases are one of the most significant global health concerns, ranking amongst the leading causes of morbidity and mortality [1,2,3]. Obesity has also reached epidemic levels globally and is a primary driver of metabolic syndrome. One of the main culprits is visceral adipose tissue (VAT), a major contributor to energy homeostasis and endocrine signaling, involved in regulating tissue function through the secretion of adipokines, as well as being a provider of pro-inflammatory cytokines (increased levels of TNF-α, IL-1β, and IL-6) [4,5,6]. These mediators have been shown to disrupt metabolic processes by facilitating tumorigenesis, insulin resistance, and hyperglycemia, linking obesity to chronic low-grade systemic inflammation [7,8]. TNF-α is involved in liver lipogenesis, inhibits lipid metabolism, and contributes to oxidative stress and inflammation, partly initiated by NF-κB signal transduction [9]. Similarly, IL-1β increases inflammatory cascades and induces secondary cytokines such as IL-6 upon activation [10]. Inflammation and oxidative stress together form a self-reinforcing positive feedback loop, thereby significantly advancing the pathogenesis of metabolic syndrome, clinically defined as hyperglycemia, dyslipidemia, and hepatic steatosis [11,12].

Oxidative stress is a prime pathogenic mechanism underlying hyperlipidemia and liver steatosis, leading to mitochondrial dysfunction, lipid peroxidation, and hepatocyte injury, which has helped to draw significant attention to antioxidant signaling pathways as a novel therapeutic target [13,14]. One of the most studied sites has recently become the nuclear factor erythroid 2-related factor 2 (Nrf2)/heme oxygenase-1 (HO-1) pathway. Nrf2 is a master transcription factor that regulates the cellular antioxidant response by regulating genes involved in redox balance, inflammation, and lipid metabolism. Under basal conditions, Nrf2 is held in the cytosol by Kelch-like ECH-associated protein 1 (Keap1), which ultimately subjects Nrf2 to ubiquitination and degradation [15,16,17]. Under conditions of oxidative stress, Nrf2 detaches from its repressor Keap1, migrates into the nucleus, and binds to antioxidant response elements (AREs) located within the promoters of cytoprotective genes, thereby initiating their transcription. Among the principal targets of Nrf2 is heme oxygenase-1 (HO-1), which mediates the breakdown of heme into biliverdin, carbon monoxide (CO), and free iron. These metabolites collectively exhibit antioxidant, anti-inflammatory, and cytoprotective activities [18,19]. Growing evidence supports that the Nrf2/HO-1 signaling axis is central to the pathophysiology of metabolic conditions. In obesity and fatty liver disease, defective Nrf2 activation contributes to lipid peroxidation, ROS accumulation, and mitochondrial damage [13,20,21].On the other hand, Nrf2 activation enhances the expression of antioxidants like HO-1, superoxide dismutase (SOD), and glutathione peroxidase (GPX) and leads to rapid scavenging of reactive oxygen species and lipid peroxides [22]. Additionally, Nrf2 regulates inflammatory cytokines by inhibiting their transcription (TNF-α, IL-1β, and IL-6) to diminish inflammation [23,24]. The role of Nrf2 in lipid metabolism has been well defined, and Nrf2-deficient mice exhibited compromised β-oxidation, increased liver triglyceride accumulation, and intensified effects of metabolic-associated fatty liver disease (MAFLD) [25,26,27]. Preclinical research indicates that pharmacologic activation of the Nrf2/HO-1 signaling axis shows enhanced insulin sensitivity, reduces hepatic steatosis and inflammation [28,29]. Additional benefits of activating the Nrf2/HO-1 axis include enhancing glutathione synthesis and catalase activity, as well as decreasing ROS production and maintaining mitochondrial and cellular integrity in metabolically active tissues [30,31]. Although there is significant promise in the Nrf2/HO-1 signaling pathway, there has not been a full translation of Nrf2/HO-1 modifiers to clinical application due to concerns [32].

The current medication therapy options for hyperlipidemia and fatty liver disease are statins, a class of drugs used for lipid-lowering therapy. Statins lower cholesterol by inhibiting hydroxymethylglutaryl-CoA (HMG-CoA) reductase, the rate-limiting enzyme in cholesterol biosynthesis [33,34]. In addition to their cholesterol-lowering activity, statins exert pleiotropic actions characterized by antioxidant, anti-inflammatory, and endothelial-protective effects [35,36]. Mechanistic studies, to some extent, have indicated that some of the ameliorative effects of statins can be induced via activation of the Nrf2/HO-1 pathway, with reports of enhanced DNA-binding ability of the Nrf2 factor and induction of HO-1 and GPX [37]. Additionally, the positive effects of statins can be context-dependent. For example, in a nephrotoxicity model, atorvastatin failed to reduce oxidative stress markers [38]. Furthermore, long-term statin therapy can increase the risk of type 2 diabetes, which may be mediated through coenzyme Q10 depletion, oxidative stress, and mitochondrial dysfunction [39,40,41].

Concurrently, glucagon-like peptide-1 (GLP-1) receptor agonists, such as liraglutide, have gained attention as promising therapeutic agents. GLP-1, which is secreted by enteroendocrine L-cells of the intestine, contributes to glycemic regulation by enhancing insulin release, slowing gastric emptying, and reducing appetite. Clinically, liraglutide is used both as an antidiabetic and as an adjunct for weight loss in the obese population [42,43]. In addition to metabolic advantages, liraglutide also shows beneficial effects on the lipid profile [44]. It demonstrates both antioxidant and anti-inflammatory effects, in part via modulation of Nrf2 and protection from obesity induced fatty infiltration of the liver [13,45].

Collectively, current findings indicate that the modulation of the Nrf2/HO-1 pathway represents a common mechanism through which both statins and GLP-1 receptor agonists mitigate oxidative stress and inflammatory processes. However, additional work is still needed to determine the molecular and comparative differences between these agents in treating hyperlipidemia and hyperechoic fatty liver. Given the aforementioned potential benefits, the purpose of this study was to examine how liraglutide impacted the effects of atorvastatin on obesity, hyperlipidemia, and fatty liver induced by a high-fat diet (HFD) in the rat. Also of interest was how liraglutide could affect the anti-inflammatory and antioxidant effects of atorvastatin, specifically via the Nrf2/HO-1 signaling pathway, in adipose tissue and the liver.

## 2. Materials and Methods

This study was carried out in the Department of Pharmacology and the Medical Research Center, Faculty of Medicine, Ain Shams University. All experimental procedures were performed in strict accordance with the institutional guidelines for animal care and were approved by the Ethics Committee for Scientific Research, Faculty of Medicine, Ain Shams University. The local research committee of Ain Shams University, Cairo, Egypt, accepted the study (Approval code: FMASUR2302022). The results were reported in accordance with the ARRIVE and BJP criteria.

### 2.1. Experimental Protocol

Fifty adult male Wistar rats weighing 250–300 gm were purchased from the National Research Institute (Cairo, Egypt). An acclimatization period of one week is required before initiating the experimental procedures. The animals were housed under a 12 h light/dark cycle at 23 ± 1 °C, with free access to food and water. Rats were maintained in cages measuring 41 × 34 × 16 cm (two animals per cage) and were carefully handled prior to experimentation to minimize stress.

### 2.2. Chemicals and Drugs

Rat chow standard diet (20% proteins, 10% fat, and 70% carbohydrates) was sourced from (Meladco, Aubor City, Cairo, Egypt) for Animal Food. Conversely, the HFD, composed of 72.5% standard rat chow supplemented with 2% cholesterol, 0.2% bile salts, and 25% commercial lard, was also sourced from the same supplier (Meladco, Aubor City, Cairo, Egypt) [46]. Liraglutide (Victoza^®^), a prefilled pen purchased from (Novo Nordisk S.p.A, Rome, Italy) with 18 mg liraglutide in 3 mL solution, was injected subcutaneously (s.c.) at a concentration of 0.8 mg/kg/day [47]. Atorvastatin was administered orally in a concentration of 10 mg/kg suspension daily for a period of 9 weeks 33. Streptozotocin (STZ) was obtained from Sigma-Aldrich (Louis, MO, USA) and freshly dissolved in 0.1 M citrate buffer (pH 4.5) immediately before injection. All drug solutions were prepared freshly just prior to administration.

### 2.3. Animals and Grouping

Rats were allocated into 2 primary groups: Group I (*n* = 10) (ND group): rats administered a normal chow diet (ND), rats fed a normal chow diet and supplemented with Saline 0.9% intraperitoneally (i.p.) in a volume of 0.1 mL/100 g body. Group II (*n* = 40) high-fat diet (HFD) group: rats fed HFD for 12 weeks: Group II a (*n* = 10): HFD group/saline, Group II b (*n* = 10): HFD group/Atorvastatin, Group II c (*n* = 10): HFD group/liraglutide, and Group II d (*n* = 10): HFD group/Atorvastatin + liraglutide

### 2.4. Induction of Diabetes

The initial body weight was recorded after acclimation of fifty western rats for one week. Rats were randomly allocated into two primary groups: the first group (ND) (*n* = 10) was fed a normal chow diet, and the second group (*n* = 40) was fed HFD for 14 days. The first group (ND) received 1 mL/kg citrate buffer (pH 4.4) (i.p.) Meanwhile, the second group of rats received i.p. STZ (35 mg/kg). Serum glucose was recorded. After 3 weeks, diabetic rats with fasting serum glucose levels ≥300 mg/dL were selected for investigation [48].

### 2.5. Drugs Treatment

Rats were assigned to their groups. Liraglutide and Atorvastatin were administered daily for 9 weeks after STZ diabetes induction. After the study’s completion, body weight was recorded, blood samples were collected from the lateral tail vein, and centrifuged serum was isolated and frozen at −80 °C. Then, rats were sacrificed by decapitation. Liver and adipose tissue were isolated and frozen at −80 °C until subsequent biochemical analysis. Part of the liver sample was dissected and kept in 10% formalin for histological investigations. Liver tissue sections were examined using an Olympus CKX41 light microscope (Olympus Corporation, Tokyo, Japan) to evaluate potential histopathological alterations.

### 2.6. Biochemical Measurement

i. Assessment of fasting serum glucose level and HbA1C

Glycosylated hemoglobin levels (HbA1C) were assessed following the procedure designated by Indan et al., 1980 [49] using the glucose Calorimetric PAP Detection Kit (Greiner Diagnostic GmbH, Bahlingen, Germany). Fasting sugar level was calorimetrically estimated using (CAT. # 81693, Crystal Chem Inc., Downers Grove, IL, USA).

ii. Assessment of Serum lipid profile

Serum lipid profile, including total cholesterol (MyBioSource, San Diego, CA, USA; Cat. No. MBS722885), triglycerides (MyBioSource, San Diego, CA, USA; Cat. No. MBS726298), high-density lipoprotein (HDL; Abcam, Waltham, MA, USA/Branford, CT, USA; Cat. No. ab65390), and low-density lipoprotein (LDL) (LDL; BioMatik, BioMatik, Kitchener, ON, Canada Cat. No. EKC39353-96T), was quantified using commercial ELISA kits according to the manufacturers’ protocols.

iii. Assessment of Oxidative stress in serum and Liver tissue.

Reduced glutathione (GSH) (Cat. # orb782371, Biorbyt Ltd., Cambridge, UK), malondialdehyde (MDA) (Cat. # orb567885; Biorbyt Ltd., Cambridge, UK), and superoxide dismutase (SOD) (Cat. # orb782014; Biorbyt Ltd., Cambridge, UK) were quantified using Enzyme-linked immunosorbent assay in serum and liver tissue. The experimental procedures were carried out in strict accordance with the manufacturer’s protocols and complied fully with established guidelines.

iv. Assessment of inflammatory markers in serum and Liver tissue

Tumor necrosis factor alpha (TNF-α) (Cat. # KRC3011; Thermo Fisher Scientific, Inc., Waltham, MA, USA), and interleukin 1 (IL-1) were determined using an enzyme-linked immunosorbent assay for serum and liver (50 mg. The concentrations were quantified according to the manufacturer’s instructions. Of those, only five have been thoroughly studied.

v. Glutathione Peroxidase (GPx) Activity Assay

Glutathione peroxidase (GPx) activity was assayed using the method mentioned [50]. The assay quantifies the rate of oxidation of reduced glutathione, measured indirectly through the decrease in NADPH concentration catalyzed by glutathione reductase. The enzyme activity was expressed in units per mg protein.

vi. Measurement of Adiponectin and Leptin Levels

Blood samples were used to measure both serum adiponectin and leptin. Adiponectin concentration was determined by the ELISA method using Sigma-Aldrich (RAB1136): Rat Adiponectin ELISA Kit, typically 96-well, with sensitivity < 0.8 ng/mL. Serum leptin concentration was determined by the ELISA method using Sigma-Aldrich (EZRL-83K): Rat Leptin ELISA Kit (Millipore), typically 96-well, sensitive from 0.05 ng/mL (https://pmc.ncbi.nlm.nih.gov/articles/PMC12010986/, accessed on 23 March 2026).

### 2.7. Molecular Study

#### 2.7.1. Assessment of Liver and Adipose Tissue NrF2, HO-1 Expression

Total RNA was isolated from homogenized liver and adipose tissue samples using TRIzol^®^ reagent (Invitrogen, Carlsbad, CA, USA), following the manufacturer’s recommended protocol. RNA concentration and purity were assessed spectrophotometrically (SmartSpec™ Plus, Bio-Rad Laboratories, Inc., Hercules, CA, USA) by measuring the absorbance at 260 nm and 280 nm, with sample integrity evaluated via the OD260/OD280 ratio. Complementary DNA (cDNA) was synthesized from total RNA using a reverse transcription kit (Promega, Madison, WI, USA), strictly adhering to the manufacturer’s instructions. Quantitative real-time PCR (qPCR) was performed on an Applied Biosystems StepOne™ Real-Time PCR System (Foster City, CA, USA) using software version 3.1. Amplification data were analyzed with PE Biosystems Sequence Detection Software v1.7. Gene-specific primers for Nrf2, HO-1, and c-Fos are detailed in Table 1.

#### 2.7.2. Semi-Quantitative PCR Analysis of IC3 and p62 Expression

Semi-quantitative PCR for IC3 and p62 expression was conducted with a One Step RT-PCR Kit (Bioline, London, UK) and GAPDH as the housekeeping gene. Agarose gel electrophoresis was performed on the PCR products, and bands were visualized using a BluePad Detection System. Band intensities were analyzed using ImageJ 1.54k [51].

#### 2.7.3. Quantitative RT-PCR Analysis of COX-2 Gene Expression

To study COX-2 gene expression, liver tissues were placed in RNAlater. Total RNA was isolated from tissue samples using TRIzol^®^ reagent (Ambion, Austin, TX, USA), in strict accordance with the manufacturer’s protocol. RNA concentration and purity were assessed using a NanoDrop 2000 spectrophotometer (Thermo Scientific, Wilmington, DE, USA), with sample quality confirmed by A260/A280 absorbance ratios. For cDNA synthesis, up to 2 μg of total RNA was reverse transcribed using oligo (dT) primers (Promega, Madison, WI, USA) under conditions specified by the manufacturer. Quantitative real-time PCR (qPCR) was performed on an Eco Real-Time PCR System (Illumina, San Diego, CA, USA) using SYBR^®^ Green qPCR Master Mix (Kapa Biosystems, Inc., Wilmington, MA, USA) and gene-specific primers (sequences provided in Table 1). Glyceraldehyde-3-phosphate dehydrogenase (GAPDH) served as the endogenous reference gene for normalization. Relative mRNA expression levels were calculated using the comparative ΔΔCT method [52].

### 2.8. Histological Study

Once liver samples were acquired from all the rats, they were subjected to a dissection process to place the samples in a 10% formalin solution. After fixation, samples were exposed to a graded percentage of 100% ethanol, dehydrated, and transformed to paraffin wax blocks for 1 h at 60 ° C, allowing paraffin block creation. The blocks were trimmed and thin-sectioned at 4 μm thickness. Some slices were stained with hematoxylin and eosin (H&E) while others were stained with Masson’s trichrome method to detect histologic changes. All sections were evaluated and photographed by light microscopy (Olympus 268 M microscope) to assess histological changes. All histological changes were evaluated in five randomly selected fields in all sections, but not consecutively.

### 2.9. Statistical Analysis

Data are presented as mean ± standard deviation (SD). GraphPad Prism (8.0.0).was used for statistical analyses. One-way analysis of variance (ANOVA) was utilized to compare the groups. Significant differences between groups were determined using Tukey’s post hoc test, for multiple comparisons, Significance: * *p* < 0.05, ** *p* < 0.01, *** *p* < 0.001, **** *p* < 0.0001 (one-way ANOVA followed by Tukey’s post hoc test). Red symbols indicate significance compared to the ND group; blue symbols indicate significance compared to the HFD group; black symbols indicate significance compared to HFD treated with ATV or Liraglutide; green symbols indicate significance compared to HFD treated with ATV or Liraglutide (*p* < 0.001). Data are expressed as mean ± SD; *n* = 6 animals per group.

### 2.10. Molecular Docking Analysis

To investigate potential molecular interactions, in silico docking simulations were performed to assess the binding affinities and interaction profiles of atorvastatin and liraglutide with two key antioxidant pathway proteins: nuclear factor erythroid 2-related factor 2 (Nrf2; PDB ID: 5CGJ) and heme oxygenase-1 (HO-1; PDB ID: 1N3U). The receptor structures were retrieved from the Protein Data Bank (https://www.rcsb.org/, accessed on 23 March 2026) and prepared by removing crystallographic water molecules, adding polar hydrogens, and optimizing protonation states. For small-molecule docking, atorvastatin was obtained from PubChem (https://pubchem.ncbi.nlm.nih.gov/compound/Atorvastatin, accessed on 23 March 2026), converted into PDB format, and energy-minimized using the MMFF94 force field in Avogadro 2.0 [53]. Both proteins and ligand structures were converted into the PDBQT format using AutoDock Tools (ADT 1.5.7), with Gasteiger charges assigned accordingly [54].

Docking of atorvastatin with Nrf2 and HO-1 was conducted using AutoDock4.2 with the Lamarckian Genetic Algorithm [55]. Grid maps were generated around the active sites identified from co-crystal structures, with a grid spacing of 0.375 Å. Each ligand–protein docking simulation was conducted with 100 independent runs to ensure thorough conformational sampling. The genetic algorithm was configured with a population size of 150 and a maximum of 2.5 × 10^6^ energy evaluations per run to optimize convergence. Resulting ligand poses were subsequently clustered based on pairwise root-mean-square deviation (RMSD) with a tolerance threshold of 2.0 Å. The cluster exhibiting the highest population density and the most favorable (lowest) predicted binding energy was selected to represent the optimal binding conformation for downstream analysis. For peptide–protein docking, liraglutide was modeled as a separate chain against the same two proteins (Nrf2 and HO-1) using AlphaFold-Multimer [56]. Five structural models were generated per complex, followed by Amber relaxation to minimize structural strain. The structural reliability of the predicted models was evaluated using the predicted Local Distance Difference Test (pLDDT) per-residue confidence scores and Predicted Aligned Error (PAE) matrices. Particular emphasis was placed on local confidence estimates at the ligand-binding interface to ensure accuracy in regions critical for molecular interaction analysis. The top-ranked models, defined by high structural reliability at the peptide–protein interface, were selected for subsequent analysis.

Visualization and interaction analyses were carried out separately for the two docking strategies. For small-molecule docking (atorvastatin–protein complexes), two-dimensional and three-dimensional binding interactions were analyzed and visualized using BIOVIA Discovery Studio Visualizer 2020, including hydrogen bonding, hydrophobic contacts, and π-interactions. For peptide–protein docking (liraglutide–protein complexes), structural inspection and binding interface analysis were performed using UCSF ChimeraX (version 1.9) and PyMol (version 2.5.7), which enabled detailed visualization of peptide orientation and receptor binding contacts [57].

## 3. Results

### 3.1. The Effect of Atorvastatin and Liraglutide on Body Weights

By investigates the impact of atorvastatin and liraglutide, alone and in combination, on body weight progression in rats fed a high-fat diet. Table 2 shows the increase in body weight of the normal diet (ND) group rats from 157 ± 4.5 g in week 1 to 260 g in week 12. The body weight of the high-fat diet (HFD) group rats increased more than that of the ND group, increasing from 155 ± 5.2 g to 316 g, which was significantly higher than that of the ND group. Rats on the atorvastatin treatment after consuming the HFD increased weight from 156 ± 4.8 g to 320 g, suggesting that the atorvastatin had no real effect on body weight. Rats that were treated with liraglutide while consuming the HFD showed a marked reduction from baseline in weight gain, as their weight increased from 152 ± 6.3 g at baseline to only 240 g at week 12, which was a greater and statistically significant reduction when compared to the untreated HFD group and the HFD/atorvastatin group (*p* < 0.0001). Finally, the atorvastatin and liraglutide combination treatment group had the lowest body weight increase (and therefore the greatest reduction in weight gain) from 155 ± 5.1 g to only 224 g, when compared to both the HFD and the HFD/atorvastatin groups; this reduction was highly significant (*p* < 0.0001).

### 3.2. The Effect of Atorvastatin and Liraglutide on Relative mRNA Expression of Nrf2 and Heme Oxygenase-1 in Liver and Adipose Tissue

**Figure 1:**
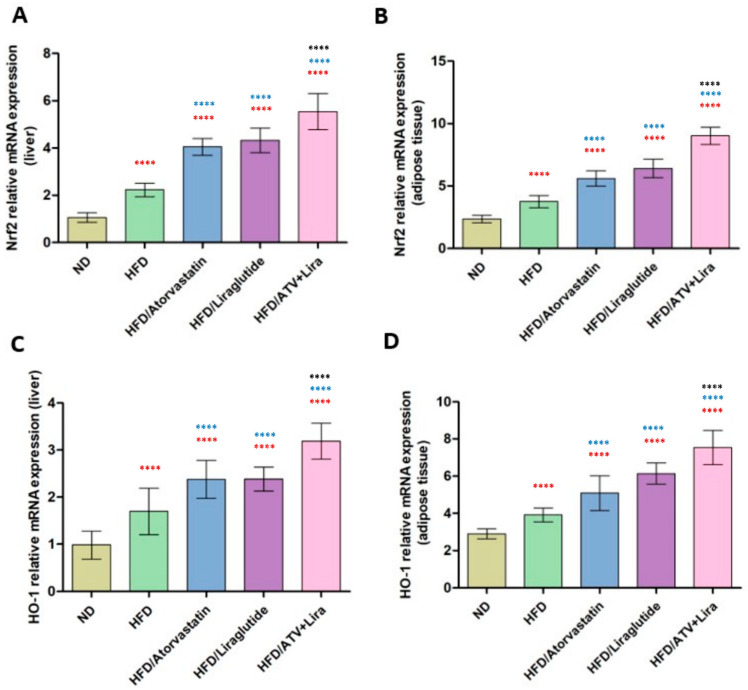
The effect of atorvastatin and liraglutide on relative mRNA expression of Nrf2 (**A**,**B**) and heme oxygenase-1 (**C**,**D**) in liver and adipose tissue, respectively. Significance: **** *p* < 0.0001 (one-way ANOVA followed by Tukey’s post hoc test). Red symbols indicate significance compared to the ND group; blue symbols indicate significance compared to the HFD group; black symbols indicate significance compared to HFD treated with ATV or Liraglutide. Data are expressed as mean ± SD; *n* = 6 animals per group. ATV: atorvastatin; Lira: liraglutide.

To determine how atorvastatin and liraglutide, alone or in combination, affect the relative mRNA expression of Nrf2 and HO-1 in liver and adipose tissues. Given the central role of the Nrf2/HO-1 signaling pathway in antioxidant defense and inflammation control, we aimed to determine whether liraglutide and atorvastatin, alone or in combination, modulate Nrf2 and HO-1 transcription in a tissue-specific manner and whether their combination exerts synergistic upregulation beyond monotherapy. As shown in Figure 1 and validated by quantitative data, 12 weeks of HFD feeding upregulated Nrf2 and HO-1 mRNA expression in hepatic and adipose tissue compared with the ND group. For the liver, mean Nrf2 expression was (1.0 ± 0.2 (ND)) and (2.3 ± 0.3 (HFD)) while HO-1 was (1.0 ± 0.3) and (2.1 ± 0.6). For adipose tissue, Nrf2 was (2.4 ± 0.3 (ND)) and (3.7 ± 0.5 (HFD)) while HO-1 was (2.9 ± 0.3 (ND)) and (3.9 ± 0.4 (HFD)). The atorvastatin or liraglutide groups further increased Nrf2 and HO-1 expression levels in comparison to the HFD group alone. For the liver, Nrf2 was 4.0 (ATV) and 4.1 (liraglutide), and HO-1 was 2.4 (ATV) and 2.5 (liraglutide). For adipose tissue, atorvastatin was Nrf2 5.2 and HO-1 4.9, whereas liraglutide for Nrf2 was 6.2 and for HO-1 was 5.8. Interestingly, the ATV + liraglutide-treated group exhibited the most substantial upregulation. Hepatic Nrf2 was 5.6, and HO-1 was 3.2; adipose tissue Nrf2 was 8.6, and HO-1 was 7.3, all statistically significant above either monotherapy or the HFD group alone (*p* < 0.0001). These findings collectively indicate a synergistic pharmacological interaction between atorvastatin and liraglutide, resulting in enhanced activation of the Nrf2/HO-1 antioxidant signaling pathway in both hepatic and adipose tissues.

### 3.3. The Effect of Atorvastatin and Liraglutide on Serum and Hepatic Levels of SOD, GSH, and MDA

**Figure 2:**
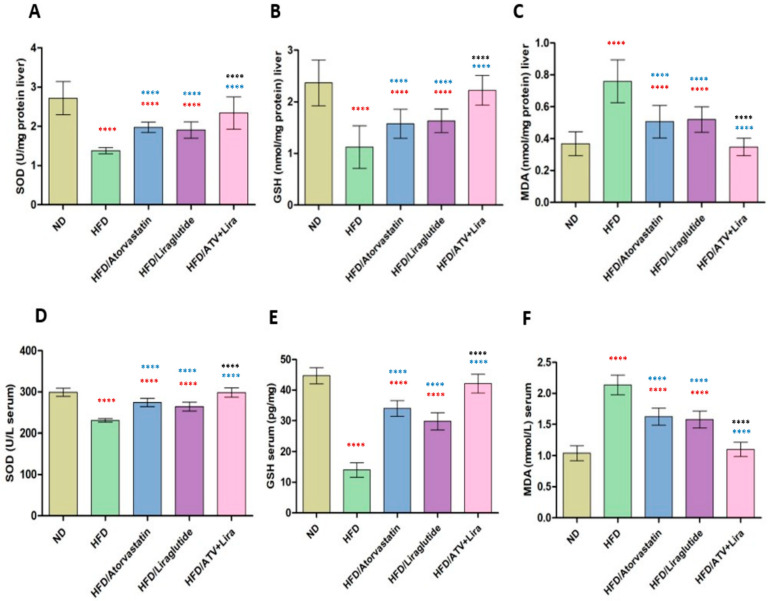
The effect of atorvastatin and liraglutide on hepatic (**A**–**C**) and serum (**D**–**F**) levels of SOD, GSH, and MDA, respectively. Significance: **** *p* < 0.0001 (one-way ANOVA followed by Tukey’s post hoc test). Red symbols indicate significance compared to the ND group; blue symbols indicate significance compared to the HFD group; black symbols indicate significance compared to HFD treated with ATV or Liraglutide. Data are expressed as mean ± SD; *n* = 6 animals per group. ATV: atorvastatin; Lira: liraglutide.

To evaluate the effect of atorvastatin and liraglutide, alone and in combination, on oxidative stress markers (SOD, GSH, and MDA) in serum and hepatic tissue of HFD-fed rats. Rats assigned to the HFD feeding group had a clear oxidative imbalance relative to the ND control, as shown in Figure 2. Evidently, serum and hepatic SOD and GSH values were significantly decreased in the HFD group (*p* < 0.0001), while MDA levels were significantly increased. For instance, hepatic SOD decreased from 2.77 ± 0.4 U/mg (ND) to 1.39 ± 0.3 U/mg (HFD), and serum SOD decreased from 299 ± 9 U/L (ND) to 232 ± 5 U/L (HFD). Likewise, hepatic GSH decreased from 2.4 ± 0.5 nmol/mg (ND) to 1.2 ± 0.3 nmol/mg (HFD); hepatic MDA increased from 0.36 ± 0.07 nmol/mg (ND) to 0.78 ± 0.1 nmol/mg (HFD); and serum MDA increased from 1.04 ± 0.1 mmol/L (ND) to 2.12 ± 0.15 mmol/L (HFD). Atorvastatin and liraglutide alone partially restored antioxidant defenses as evidenced by significant increases in SOD and GSH levels and significant reductions in MDA levels relative to the HFD group alone (*p* < 0.0001). The serum SOD was increased due to atorvastatin to 276, and the serum SOD increased due to liraglutide to 265, while hepatic GSH improved to 1.7–1.8. The hepatic and serum MDA levels were significantly decreased by atorvastatin or liraglutide therapy alone compared to the HFD.

The Atorvastatin-liraglutide cotherapy resulted in the most notable antioxidant effect. Serum SOD was restored to 300 U/L, and hepatic SOD was restored to 2.2–2.5 U/mg, or similar to ND controls. GSH levels were also returned to normalized values (liver 2.3 approaching ND), and MDA levels in both serum (1.05 mmol/L) and liver (0.35–0.4 nmol/mg) again approached normal values with no significant difference to ND controls. These results showed that both atorvastatin and liraglutide individually enhance redox balance, but in combination, they produce a synergistic effect that nearly completely reverses HFD-induced oxidative stress.

### 3.4. The Effect of Atorvastatin and Liraglutide on Serum Lipid Profile

**Figure 3:**
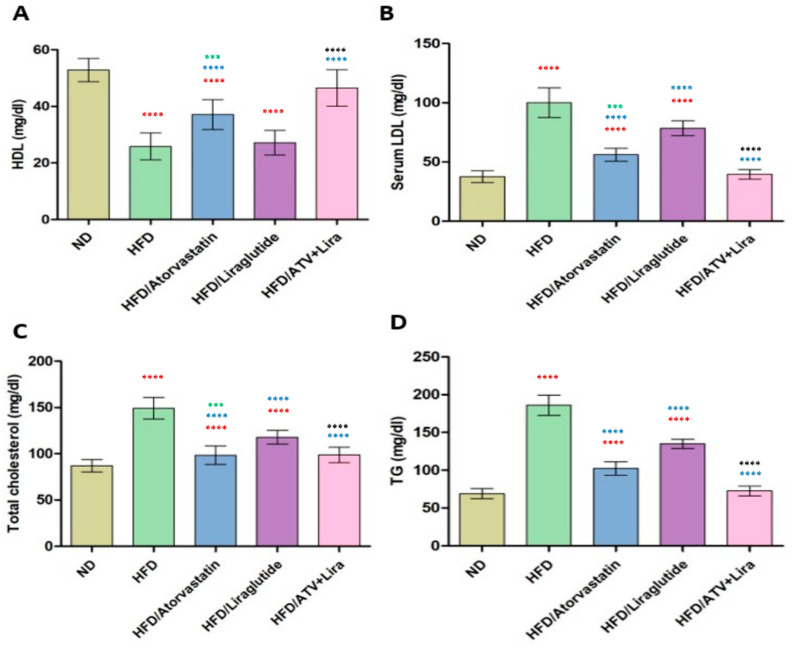
The effect of atorvastatin and liraglutide on serum lipid profile of HDL (**A**), serum LDL (**B**), Total cholesterol (**C**), and TG (**D**). Significance: *** *p* < 0.001, **** *p* < 0.0001 (one-way ANOVA followed by Tukey’s post hoc test). Red symbols indicate significance compared to the ND group; blue symbols indicate significance compared to the HFD group; black symbols indicate significance compared to HFD treated with ATV or Liraglutide; green symbols indicate significance compared to HFD treated with ATV or Liraglutide (*p* < 0.001). Data are expressed as mean ± SD; *n* = 6 animals per group. ATV: atorvastatin; Lira: liraglutide.

To evaluate the individual and combined effects of atorvastatin and liraglutide on serum lipid parameters and to determine whether their combination provides enhanced improvement of HFD-induced dyslipidemia. Rats fed HFD exhibited considerable dyslipidemia, characterized by significant increases in triglycerides (TG), total cholesterol (TC), and low-density lipoprotein-cholesterol (LDL), along with significant decreases in high-density lipoprotein-cholesterol (HDL), compared to the ND group (Figure 3). LDL increased from 39 ± 5 mg/dL (ND) to 101 ± 12 mg/dL (HFD), and HDL decreased from 52 ± 4 mg/dL (ND) to 25 ± 4 mg/dL (HFD). Liraglutide treatment produced significant improvement in lipid profiles compared to high-fat, with LDL down to 77 ± 7 mg/dL, TC to 119 ± 7 mg/dL, TG to 136 ± 5 mg/dL, and a modest increase in HDL to 27–31 mg/dL (*p* < 0.05). Atorvastatin had more lipids and cholesterols than liraglutide, but brought LDL down to 55 ± 5 mg/dL, TCs to 98 ± 9 mg/dL, TGs to 102 ± 7 mg/dL, and HDL showed a modest increase to 37 ± 4 mg/dL. Liraglutide and atorvastatin were used to maximize the changes, resulting in a nearly complete restoration of lipid parameters back to ND levels. HDL was decreased to 46 ± 6 mg/dL, LDL values decreased to 40 ± 4 mg/dL, and TC decreased to 98 ± 8 mg/dL. TGs were returned to 72 ± 6 mg/dL. Data indicate these values demonstrated no significant differences compared to the ND control group pre-mealtime high-fat intervention. The data indicate that both compounds were efficacious for dyslipidemia irrespective of lipid profile changes.

### 3.5. The Effect of Atorvastatin and Liraglutide on Serum and Hepatic Levels of Inflammatory Cytokines

**Figure 4:**
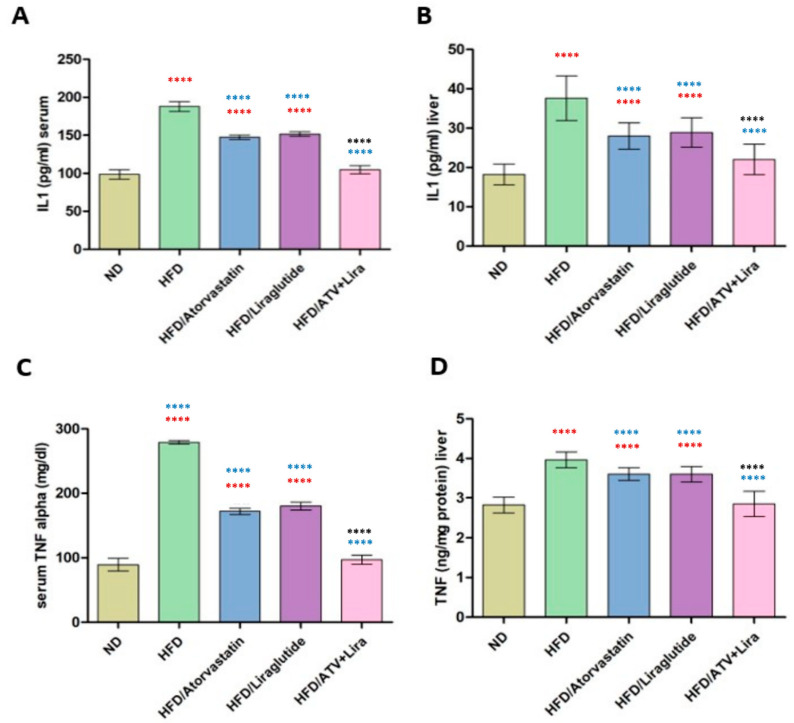
The effect of atorvastatin and liraglutide on hepatic (**A**,**B**) and serum (**C**,**D**) levels of inflammatory cytokines (IL-1β and TNF, respectively). Significance: **** *p* < 0.0001 (one-way ANOVA followed by Tukey’s post hoc test). Red symbols indicate significance compared to the ND group; blue symbols indicate significance compared to the HFD group; black symbols indicate significance compared to HFD treated with ATV or Liraglutide. Data are expressed as mean ± SD; *n* = 6 animals per group. ATV: atorvastatin; Lira: liraglutide.

To evaluate the individual and combined effects of atorvastatin and liraglutide on inflammatory cytokine levels and to determine whether their combination exerts a synergistic anti-inflammatory effect. As highlighted in Figure 4, HFD-fed rats showed a strong pro-inflammatory response, with increased hepatic and serum TNF-α and IL-1β compared to animals fed an ND. The hepatic TNF-α increased from 2.8 ± 0.2 ng/mg (ND) to 3.9 ± 0.2 ng/mg (HFD) while serum TNF-α increased substantially from 91 ± 9 mg/dL (ND) to 279 ± 3 mg/dL (HFD). IL-1β was similar, the hepatic values nearly doubled (18 ± 3 pg/mL vs 38 ± 5pg/mL), and serum IL-1β increased from 100 ± 5 pg/mL to 188 ± 6 pg/mL. Treatments with atorvastatin or liraglutide alone caused a significant decrease in all res. As previously discussed, atorvastatin treatment reduced hepatic TNF-α, which was 3.6 ± 0.2 ng/mg, and serum TNF-α, which was 170 ± 5 mg/dL. Interestingly, liraglutide had similar results (3.7 ± 0.2 mg/dL in liver, 178 ± 6 mg/dL in serum). Reduction was also seen when looking at IL-1β, where both atorvastatin and liraglutide had an average of 27–30 pg/mL in the liver and 150 pg/mL in serum. The most marked anti-inflammatory response was observed for the combination of atorvastatin and liraglutide, where serum cytokine levels returned to similar values as control (reduced hepatic TNF-α and IL-1β levels to 2.7 ng/mg and 21 pg/mL, respectively, and serum TNF-α and IL-1β levels returned to 95–105 mg/dL for TNF-α, and 105 pg/mL for IL-1β that was similar to ND). These results demonstrate that both atorvastatin and liraglutide decreased HFD-induced levels of inflammatory cytokines; however, the combined effects did more than add to reduce the levels of systemic inflammatory cytokines and the normalization of hepatic cytokine levels.

### 3.6. The Effect of Atorvastatin and Liraglutide on Serum Levels of FBG and HbA1c

**Figure 5:**
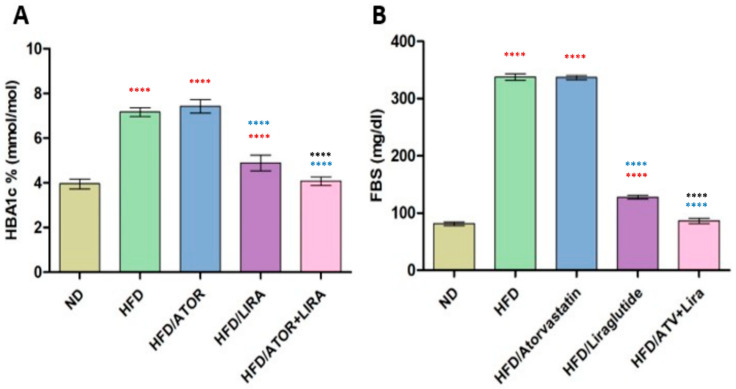
The effect of atorvastatin and liraglutide on serum HbA1c (**A**) and FBG (**B**) serum levels. Significance: **** *p* < 0.0001 (one-way ANOVA followed by Tukey’s post hoc test). Red symbols indicate significance compared to the ND group; blue symbols indicate significance compared to the HFD group; black symbols indicate significance compared to HFD treated with ATV or Liraglutide. Data are expressed as mean ± SD; *n* = 6 animals per group. ATV: atorvastatin; Lira: liraglutide.

To determine the efficacy of atorvastatin and liraglutide, individually and combined, in improving glucose homeostasis and reducing hyperglycemia in HFD-induced metabolic dysfunction (Figure 5). Rats fed HFD exhibited pronounced hyperglycemia; fasting blood glucose (FBG) levels rose significantly from 81 ± 3 mg/dL in ND (normal diet) rats to 338 ± 5 mg/dL in HFD rats (*p* < 0.05). Likewise, HbA1c levels were also markedly elevated in HFD compared to controls. Monotherapy with atorvastatin failed to improve glycemic status. FPG in atorvastatin-treated rats remained high (335 ± 4 mg/dL), and even though atorvastatin administration resulted in a numeric increase in HbA1c values compared to HFD, this increase was not significant. Liraglutide monotherapy significantly reduced FBG levels to 127 ± 3 mg/dL and HbA1c values compared to HFD controls (*p* < 0.05). Combination therapy (atorvastatin + liraglutide) produced the most pronounced effect, returning FBG values (87 ± 4 mg/dL) to a level that could not be statistically distinguished from ND rats, with HbA1c values similarly restored when compared to HFD controls. These data suggest that atorvastatin alone is unable to mediate glucose homeostasis, whereas liraglutide appears to mediate marked anti-hyperglycemic effects; in fact, the combination of liraglutide and atorvastatin resulted in a synergistic restoration of normoglycemia.

### 3.7. The Effect of Atorvastatin and Liraglutide on Adiponectin and Leptin Levels

**Figure 6:**
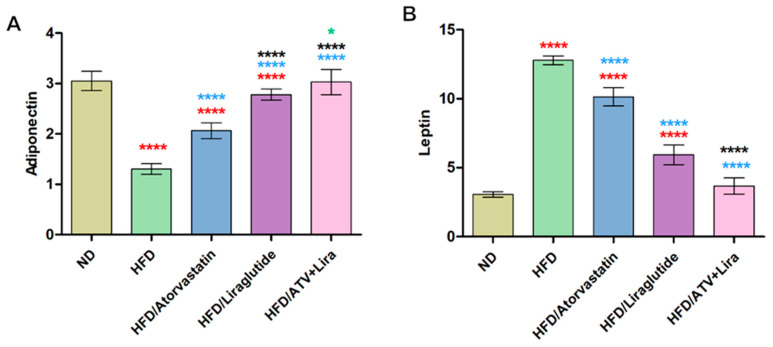
Effect of atorvastatin and liraglutide pretreatment on Adiponectin (**A**) and Leptin (**B**) levels in rat liver. Significance: * *p* < 0.05, **** *p* < 0.0001 (one-way ANOVA followed by Tukey’s post hoc test). Red symbols indicate significance compared to the ND group; blue symbols indicate significance compared to the HFD group; black symbols indicate significance compared to HFD treated with ATV; green symbols indicate significance compared to HFD treated with Liraglutide. Data are expressed as mean ± SD; *n* = 6 animals per group. ATV: atorvastatin; Lira: liraglutide.

To determine the individual and combined effects of atorvastatin and liraglutide on obesity-associated dysregulation of adiponectin and leptin, and to evaluate whether their combination synergistically restores metabolic homeostasis. A high-fat diet had a pronounced reduction in adiponectin levels, which were measured at 1.30 ng/mL, compared to the normal diet, which was 6.33 ng/mL, and a pronounced increase in leptin levels, which were measured at 12.88 ng/mL, compared to the normal diet, which was 4.87 ng/mL (Figure 6). This indicates a reduced insulin sensitivity and possible development of leptin resistance. Treatment with atorvastatin allowed for improvements; adiponectin increased to 2.06 ng/mL, and leptin decreased to 10.14 ng/mL. These levels are still significantly different than the normal group. On the other hand, liraglutide had an even more pronounced effect with an increase in adiponectin to 2.78 ng/mL and a decrease in leptin to 5.93 ng/mL, allowing for adiponectin and leptin values to approach physiological ranges. As shown in Figure 6, Adiponectin levels for the combined intervention (HFD/ATV and Lira) resulted in nearly returning to normal (3.03 ng/mL), whereas leptin levels were approaching those seen with the ND (3.67 ng/mL). Therefore, these results show that the HFD disrupts the normal balance of adipokines and that pharmacological interventions, especially combination therapy, can markedly restore metabolic homeostasis.

### 3.8. Effect of Atorvastatin and Liraglutide on Hepatic COX-2 Expression

**Figure 7:**
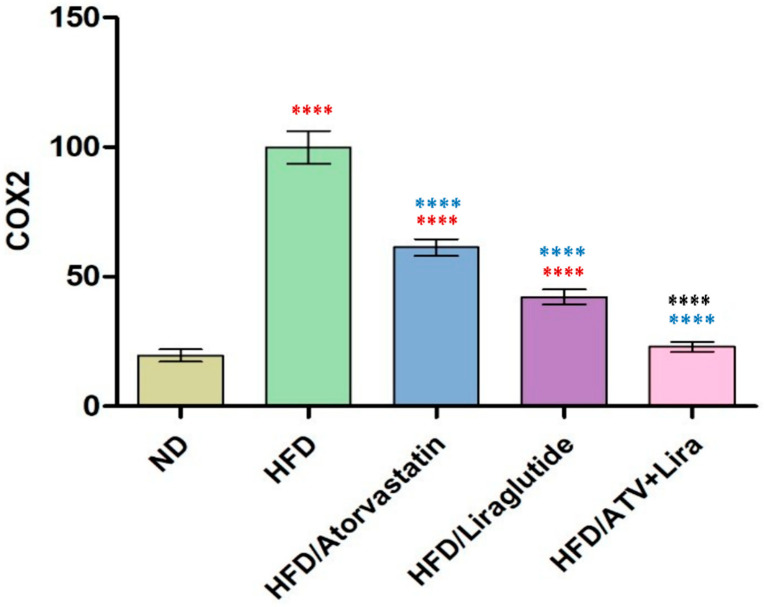
Effect of atorvastatin and liraglutide pretreatment on hepatic COX-2 expression. Significance: **** *p* < 0.0001 (one-way ANOVA followed by Tukey’s post hoc test). Red symbols indicate significance compared to the ND group; blue symbols indicate significance compared to the HFD group; black symbols indicate significance compared to HFD treated with ATV or Liraglutide. Data are expressed as mean ± SD; *n* = 6 animals per group. ATV: atorvastatin; Lira: liraglutide.

To determine the individual and combined effects of atorvastatin and liraglutide on hepatic COX-2 levels and to evaluate whether their combination synergistically reduces obesity-associated liver inflammation. Effects of different treatments on hepatic COX-2 expression were studied (Figure 7). HFD induced a significant increase in the levels of hepatic COX-2 compared with the ND (HFD: 99.9 ± 6.3; ND 19.6 ± 2.4, *p* < 0.05, as seen in Figure 6. Atorvastatin administration resulted in a significant decrease in COX-2 levels compared with the HFD group (61.3 ± 3.3), while liraglutide induced a larger decrease (42.2 ± 2.9, *p* < 0.05). Finally, administration of atorvastatin with liraglutide resulted in the greatest reduction in COX-2 levels (22.9 ± 2.0) and was close to CN.

### 3.9. The Effect of Atorvastatin and Liraglutide on LC3 and p62 Expression

**Figure 8:**
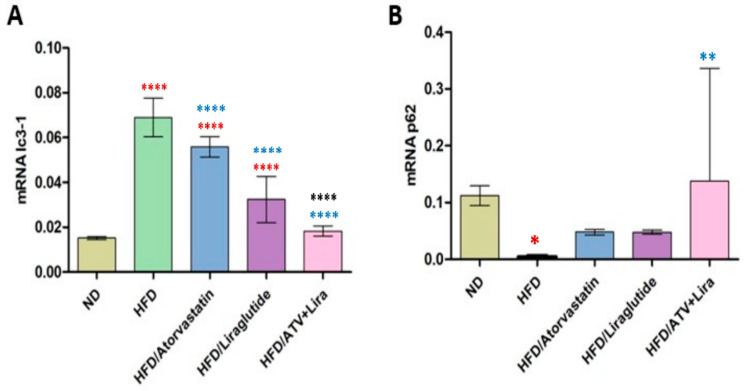
Effect of atorvastatin and liraglutide pretreatment on the expression of LC3 (**A**) and p62 (**B**) in rat liver. Significance: * *p* < 0.05, ** *p* < 0.01, **** *p* < 0.0001 (one-way ANOVA followed by Tukey’s post hoc test). Red symbols indicate significance compared to the ND group; blue symbols indicate significance compared to the HFD group; black symbols indicate significance compared to HFD treated with ATV or Liraglutide. Data are expressed as mean ± SD; *n* = 6 animals per group. ATV: atorvastatin; Lira: liraglutide.

To determine the individual and combined effects of atorvastatin and liraglutide on hepatic autophagy, we evaluated whether their combination synergistically restores LC3 and p62 expression toward normal levels in HFD-fed obese rats. The effect on LC3 and p62 expression was investigated in Figure 8, indicating that LC3 levels were increased significantly in the HFD group (0.069 ± 0.007) versus the ND group (0.015 ± 0.001). Treatment with atorvastatin (0.056 ± 0.005) and liraglutide (0.034 ± 0.010) significantly lowered LC3 compared to the HFD group. The combination of atorvastatin and liraglutide reduced LC3 further to a value of (0.019 ± 0.003), close to the ND values. Conversely, p62 was significantly lowered in the HFD group (0.004 ± 0.002) compared to ND rats (0.111 ± 0.016). Treatment with atorvastatin (0.048 ± 0.005) or liraglutide (0.048 ± 0.004) partially restores p62, and treatment with atorvastatin and liraglutide (0.079 ± 0.004) exhibited a more substantial increase, probably close to control.

### 3.10. Histological Results

**Figure 9:**
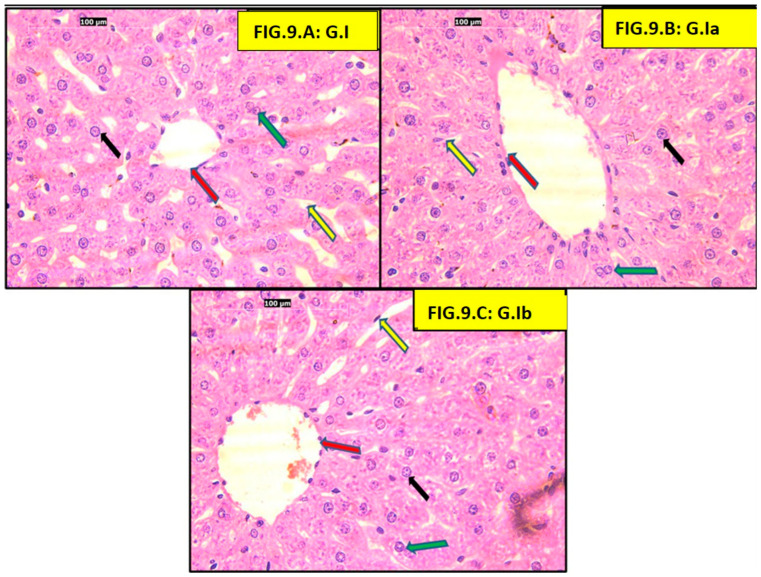
(**A**) Photomicrograph of section in G.1 control (ND) adult rat liver showing normal central vein (red ↑), normal hepatocytes with vesicular nuclei and acidophilic cytoplasm with prominent nucleolus (black ↑), arranged in radiating plates and separated by blood sinusoids lined with flat cells with flat nuclei (yellow ↑). Some hepatocytes are binucleated (green ↑). (**B**) Photomicrograph of section in G.1a positive control (ND + Atorvastatin treated) adult rat liver showing normal central vein (red ↑), normal hepatocytes with vesicular nuclei and acidophilic cytoplasm with prominent nucleolus (black ↑), arranged in radiating plates and separated by blood sinusoids lined with flat cells with flat nuclei (yellow ↑). Some hepatocytes are binucleated (green ↑). (**C**) Photomicrograph of section in G.1b positive control (ND + liraglutide treated) adult rat liver positive control adult rat liver showing normal central vein (red ↑), normal hepatocytes with vesicular nuclei and acidophilic cytoplasm with prominent nucleolus (black ↑), arranged in radiating plates and separated by blood sinusoids lined with flat cells with flat nuclei (yellow ↑) Some hepatocytes are binucleated (green ↑). (H&E ×400).

Hematoxylin and eosin (H&E) examination of both control and positive control groups showed normal liver architecture, including a central vein, hepatocytes arranged in radiating plates, and blood sinusoids. Hepatocytes in all sections had vesicular nuclei, acidophilic cytoplasm, and prominent nucleoli. Some binucleated hepatocytes were observed (Figure 9).

**Figure 10:**
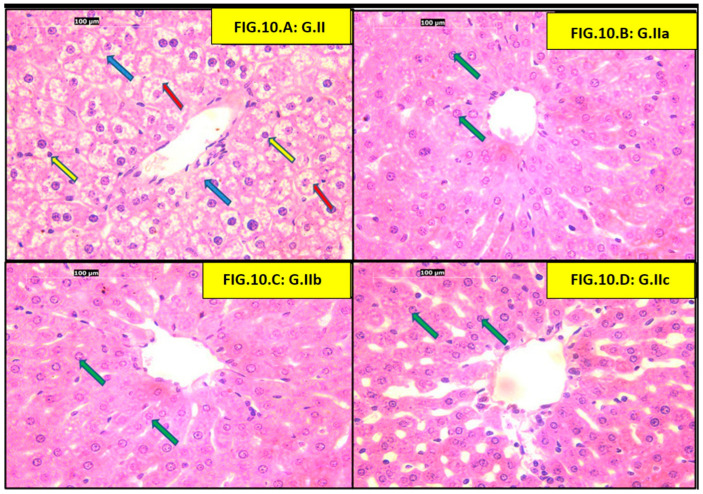
(**A**) Photomicrograph of section in G.II (HFD) adult rat liver showing Many ballooned hepatocytes (round swelling and clear cytoplasm) (red ↑) and Mallory-Denk bodies (eosinophilic intracellular inclusions; blue ↑) are seen. Yellow ↑ indicates an apoptotic body. (**B**) Photomicrograph of a section in G.IIa (HFD + Atorvastatin-treated) adult rat liver shows the treatment’s obvious protective effect against the HFD pathological effects (green ↑). (**C**) Photomicrograph of a section in G.IIb (HFD + liraglutide-treated) adult rat liver shows the treatment’s obvious protective effect against the HFD pathological effects (green ↑). (**D**) Photomicrograph of a section in G.IIc (HFD + Atorvastatin + liraglutide-treated) adult rat liver showing the obvious protective effect of the treatment against the HFD pathological effects (green ↑). (H&E ×400).

Sections of adult rat livers fed a high-fat diet (HFD) showed ballooned hepatocytes (swollen with clear cytoplasm) and Mallory-Denk bodies (eosinophilic intracellular inclusions). All three treatment groups (atorvastatin, liraglutide, and atorvastatin + liraglutide) showed a reduction in ballooned hepatocytes and Mallory-Denk bodies (Figure 10).

**Figure 11:**
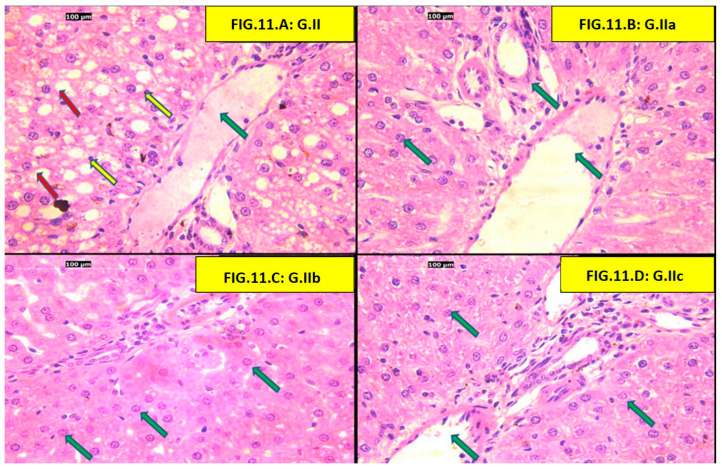
(**A**) Photomicrograph of a section in G.II (HFD) adult rat liver portal triad showing a congested portal vein (green ↑), many ballooned hepatocytes (round swelling and clear cytoplasm) (red ↑), and yellow ↑ indicates an apoptotic body. (**B**) Photomicrograph of a section in G.IIa (HFD + Atorvastatin-treated) adult rat liver shows the treatment’s obvious protective effect against the HFD pathological effects (green ↑). (**C**) Photomicrograph of a section in G.IIb (HFD + liraglutide-treated) adult rat liver showing the obvious protective effect of the treatment against the HFD pathological effects (green ↑). (**D**) Photomicrograph of a section in G.IIc (HFD + Atorvastatin + liraglutide-treated) adult rat liver showing the obvious protective effect of the treatment against the HFD pathological effects (green ↑). (H&E ×400).

Examination of the portal triad zone of the (HFD) group showed congested portal veins, ballooned hepatocytes, and apoptotic bodies, which were not evident in all three treatment groups (atorvastatin, liraglutide, and atorvastatin + liraglutide) (Figure 11).

**Figure 12:**
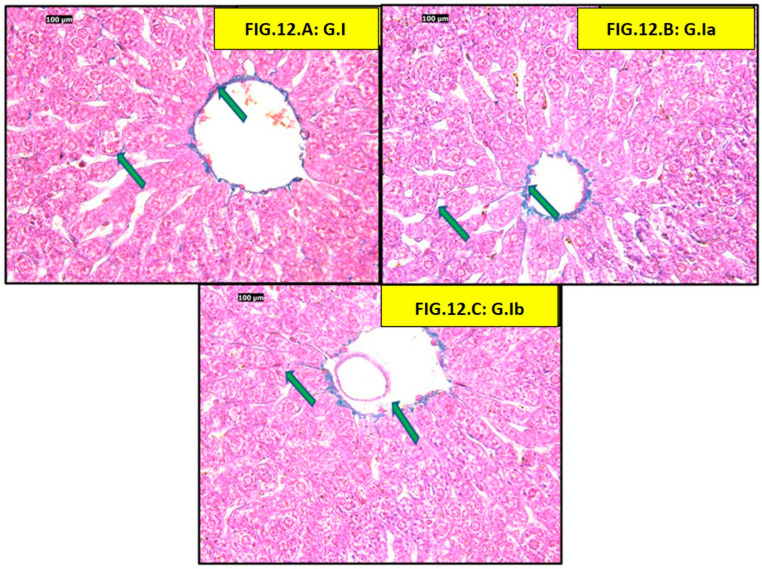
(**A**) Photomicrograph of a section in G.1 control (ND)adult rat liver showing normal architecture of the different parts of the liver tissues, with scanty normal collagen fibers distributed surrounding the central vein and in the interstitial tissue (green ↑). (**B**) Photomicrograph of section in G.1a positive control (ND + Atorvastatin treated) adult rat liver shows normal architecture of the different parts of the liver tissues, with scanty normal collagen fibers distribution surrounding the central vein and interstitial tissue (green ↑). (**C**) Photomicrograph of section in G.1b positive control (ND + liraglutide treated) adult rat liver, showing normal architecture of the different parts of the liver tissues, with scanty normal collagen fibers distribution surrounding the central vein and in the interstitial tissue (green ↑). (Masson trichrome ×400).

Masson trichrome examination of both control and positive control groups showed normal liver architecture, and normal collagen fibers were observed surrounding the central vein and interstitial tissue (Figure 12).

**Figure 13:**
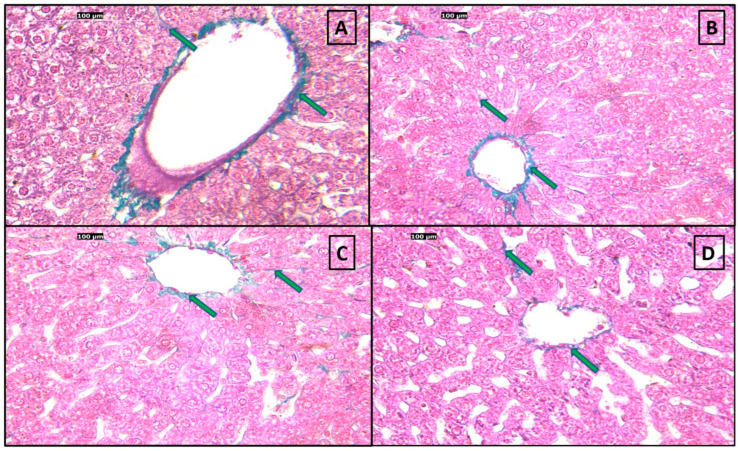
(**A**) Photomicrograph of a section in G.II (HFD) adult rat liver showing marked deposition of collagen fibers surrounding the central vein and in the interstitial tissue (green ↑). (**B**) Photomicrograph of a section in G.IIa (HFD + Atorvastatin-treated) adult rat liver shows minimal deposition of collagen fibers surrounding the central vein and in the interstitial tissue compared to the HDF group (green ↑). (**C**) Photomicrograph of a section in G.IIb (HFD + liraglutide-treated) adult rat liver shows minimal deposition of collagen fibers surrounding the central vein and in the interstitial tissue compared to the HDF group (green ↑). (**D**) Photomicrograph of a section in G.IIc (HFD + Atorvastatin + liraglutide-treated) adult rat liver showing normal architecture of the different parts of the liver tissues, with a marked decrease in collagen fiber distribution in the interstitial tissue compared to the HFD group. (Masson trichrome ×400).

The HFD group showed increased collagen deposition surrounding the central vein and interstitial tissue. (atorvastatin, liraglutide, and atorvastatin + liraglutide) Individual and combined treated groups showed reduced collagen deposition (Figure 13).

**Figure 14:**
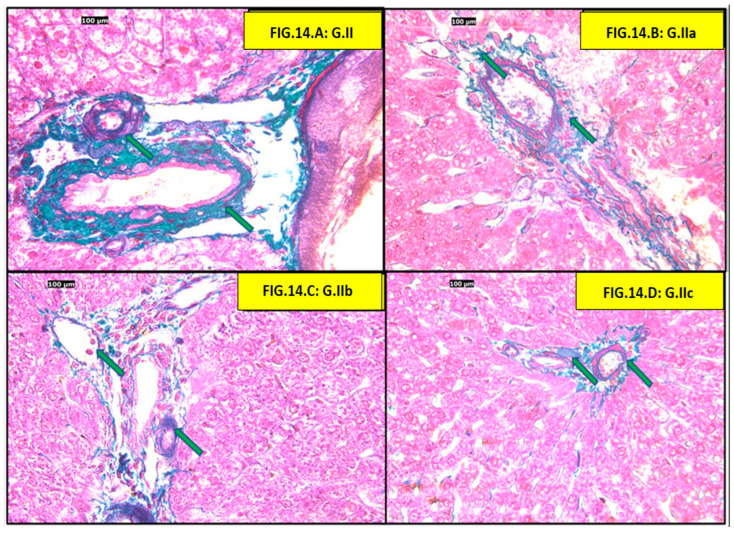
(**A**) Photomicrograph of a section in G.II (HFD) adult rat liver showing the portal area with marked deposition of collagen fibers surrounding the portal vein, the bile duct (BD), and the hepatic artery (green ↑). (**B**) Photomicrograph of a section in G.IIa (HFD + Atorvastatin-treated) adult rat liver showing the portal area with minimal deposition of collagen fibers surrounding the portal vein, bile duct (BD), and hepatic artery in comparison to the HDF group (green ↑). (**C**) Photomicrograph of a section in G.IIb (HFD + liraglutide-treated) adult rat liver showing the portal area with minimal deposition of collagen fibers surrounding the portal vein, bile duct (BD), and hepatic artery in comparison to the HDF group (green ↑). (**D**) Photomicrograph of a section in G.IIc (HFD + Atorvastatin + liraglutide-treated) adult rat liver showing portal area with minimal deposition of collagen fibers surrounding portal vein, bile duct (BD), and hepatic artery in comparison to the HDF group. (Masson trichrome ×400).

Examination of the portal triad zone HFD group showed increased collagen deposition in the portal area, surrounding the portal vein, bile duct, and hepatic artery. All three treatment groups (atorvastatin, liraglutide, and atorvastatin + liraglutide) showed reduced collagen deposition in the portal area (Figure 14).

### 3.11. Molecular Docking

The molecular docking of atorvastatin against Nrf2 (PDB ID: 5CGJ) and HO-1 (PDB ID: 1N3U) yielded favorable binding energies, indicating that the drug exhibits appreciable affinity toward both target proteins. The binding energy for atorvastatin with Nrf2 was −7.98 kcal/mol, which was slightly more favorable than that obtained with HO-1 (−7.22 kcal/mol), suggesting a stronger interaction with the Nrf2 binding pocket (Table 1).

**Figure 15:**
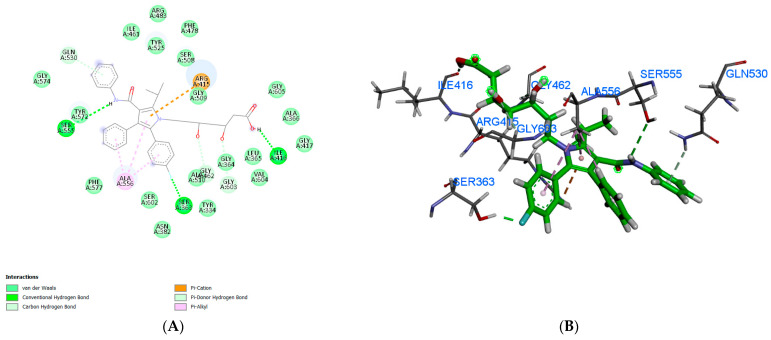

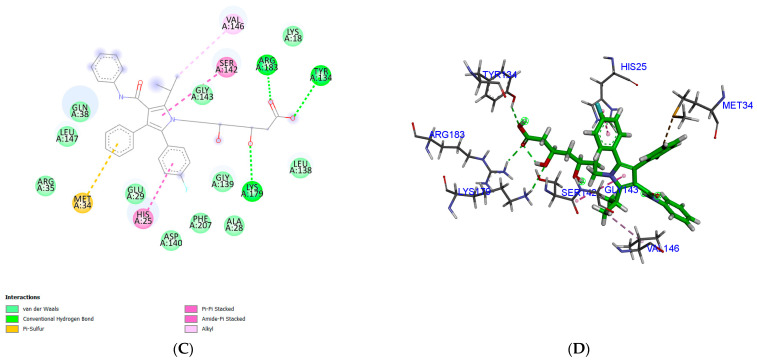
Docking interactions of atorvastatin with Nrf2 and HO-1. (**A**) Two-dimensional interaction diagram of atorvastatin with Nrf2 (PDB ID: 5CGJ), (**B**) Three-dimensional binding pose of atorvastatin within the Nrf2 active site, highlighting key interacting residues, (**C**) Two-dimensional interaction diagram of atorvastatin with HO-1 (PDB ID: 1N3U), and (**D**) Three-dimensional binding pose of atorvastatin within the HO-1 active site, highlighting key interacting residues.

For the Nrf2 complex, atorvastatin was stabilized primarily through three hydrogen bonds involving Ser363, Ile416, and Ser555, residues located within regions contributing to the protein’s regulatory function. In addition, a π-cation interaction with Arg415 was observed, further anchoring the ligand in the active site (Table 3 and Figure 15A,B). The combination of hydrogen bonding and electrostatic stabilization highlights the potential of atorvastatin to modulate Nrf2 activity by occupying critical residues within its binding cavity.

In the HO-1 complex, atorvastatin demonstrated a slightly weaker binding energy but engaged in a diverse array of noncovalent interactions that contribute to complex stabilization. The ligand formed hydrogen bonds with Tyr134, Lys179, and Arg183, residues that lie within or near the catalytic cleft of the enzyme. Moreover, several π-mediated interactions were detected: π–π stacking with His25, amide–π stacking with Ser142, and a π–sulfur interaction with Met34 (Table 3 and Figure 15C,D). These contacts provide additional stabilization and reflect the capacity of atorvastatin’s aromatic moieties to interact with the enzyme’s active-site residues.

**Figure 16:**
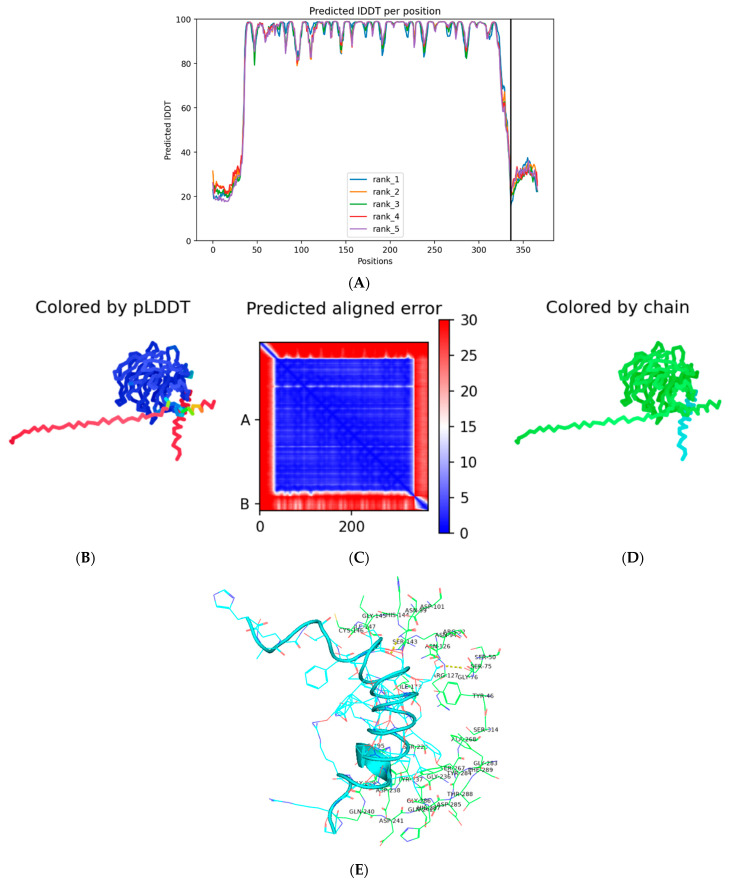
AlphaFold-Multimer docking of liraglutide with Nrf2 (PDB ID: 5CGJ). (**A**) Per-residue predicted Local Distance Difference Test (pLDDT) scores for the five predicted models, indicating local structural confidence. (**B**) Average pLDDT for the selected best model (model 3, seed 000) after three recycling iterations. (**C**) Predicted aligned error (PAE) plot showing low interface prediction error, with overall predicted TM-score (pTM = 0.818) and interface TM-score (ipTM = 0.362) where A (Aligned/Reference Residue) is the residue around which the structure is anchored for comparison and B (Error-evaluated Residue) is the residue whose position is being measured relative to A. (**D**) Three-dimensional representation of the Nrf2 protein (green) and liraglutide peptide (cyan), highlighting the binding orientation. (**E**) Close-up view of the peptide–protein interface, showing potential hydrogen bonds and polar contacts between liraglutide and Nrf2 residues near the DNA-binding and regulatory regions.

The AlphaFold-Multimer docking of liraglutide against Nrf2 (PDB ID: 5CGJ) produced high-confidence structural models, capturing key aspects of peptide–protein interaction (Figure 15). Figure 16A shows the per-residue predicted Local Distance Difference Test (pLDDT) scores for the five predicted models, with most residues approaching values near 100, indicating excellent local structural reliability. Among the models, model 3 (seed 000) after three recycling iterations was selected as the best-performing structure. In Figure 16D, Nrf2 is represented in green and the liraglutide peptide in cyan, illustrating the overall binding orientation.

The selected model exhibited an average pLDDT of 82.2 (Figure 16B), reflecting high confidence in both backbone and side-chain placements for the protein and peptide. The global predicted TM-score (pTM = 0.818) and interface TM-score (ipTM = 0.362), combined with a very low predicted aligned error (Figure 16C), indicate moderate confidence in the global assembly and the interface geometry, which is typical for flexible peptide–protein complexes.

Structurally, liraglutide adopts a conformation that aligns along a putative binding groove on the Nrf2 surface, establishing stabilizing contacts. Visual inspection in PyMOL (Figure 16E) revealed interactions with residues near the DNA-binding and regulatory domains of Nrf2, including potential hydrogen bonds and polar contacts.

**Figure 17:**
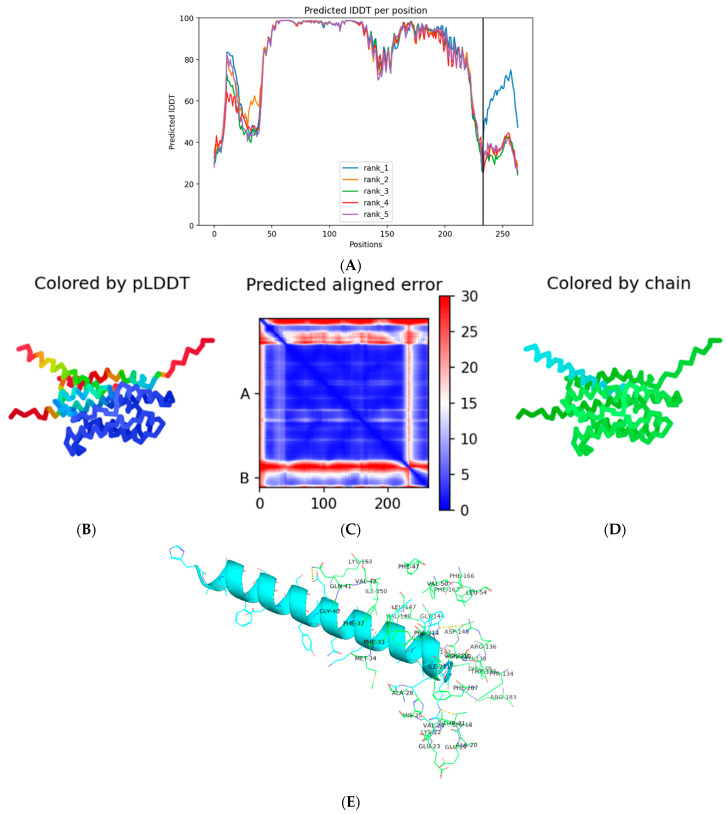
AlphaFold-Multimer docking of liraglutide with HO-1 (PDB ID: 1N3U). (**A**) Per-residue predicted Local Distance Difference Test (pLDDT) scores for the five predicted models, indicating local structural confidence. (**B**) After three recycling iterations, the average pLDDT for the selected best model (model 5, seed 000). (**C**) Predicted aligned error (PAE) plot showing low interface prediction error, with overall predicted TM-score (pTM = 0.82) and interface TM-score (ipTM = 0.619). (**D**) Three-dimensional representation of the HO-1 protein (green) and liraglutide peptide (cyan), highlighting the binding orientation. (**E**) Close-up view of the peptide–protein interface, showing potential hydrogen bonds and polar contacts between liraglutide and Nrf2 residues near the DNA-binding and regulatory regions.

Similarly, docking against HO-1 (PDB ID: 1N3U) yielded a high-confidence model, with the top-ranked structure corresponding to model 5, seed 000 (Figure 17). The model exhibited an average pLDDT of 81.4, reflecting reliable backbone and side-chain modeling. The global predicted TM-score (pTM = 0.82) and the interface predicted TM-score (ipTM = 0.619) indicate strong confidence in the overall assembly and, notably, in the peptide–protein interface (Figure 17A–C). Structurally, liraglutide fits along a putative binding groove on the HO-1 surface, forming several stabilizing contacts. Visual inspection in ChimeraX and PyMol (Figure 17D,E) highlighted potential hydrogen bonds and polar interactions with residues near the enzyme’s catalytic and regulatory regions. The high ipTM score suggests that the predicted interface is likely well-modeled, which is significant given the inherent flexibility of peptide–protein interactions.

Although AlphaFold-Multimer does not provide binding energies, the observed contacts and interface confidence suggest that liraglutide may directly engage both Nrf2 and HO-1, potentially influencing their conformational dynamics and functional accessibility. Overall, the high-confidence AlphaFold-Multimer models provide a structural rationale for liraglutide’s ability to interact with these key oxidative stress regulators, supporting its potential to enhance cytoprotective and antioxidant responses.

## 4. Discussion

Obesity, hyperlipidemia, and metabolic-associated fatty liver disease (MAFLD) are three of the most common metabolic disorders on the planet, and they impose considerable strain on health systems. These metabolic disorders are associated with chronic low-grade inflammation, oxidative stress, and dysregulated lipid metabolism, and all three conditions are collectively related to the development of metabolic syndrome and its complications [58,59,60,61]. Most of our therapeutic approaches are based on lipid-lowering medications, which require statins to be standard for treating dyslipidemia and some cardiovascular risk. The majority of clinical applications of statins show they can lower cholesterol, but they will not be able to address the more complex pieces of inflammation, oxidative stress, and the systemic dysregulation of lipid metabolism in relation to obesity [62,63,64,65]. Atorvastatin, a frequently prescribed statin, has been demonstrated to improve lipid profiles, but it can also exert anti-inflammatory and antioxidant effects. However, its usefulness is limited in obesity and MAFLD, where the mechanisms of disease progression are numerous and overlapping [62,66,67]. Liraglutide, a GLP-1 receptor agonist, has been recognized as a therapeutic agent due to its weight-reducing, insulin-sensitizing, and hepatoprotective properties. In addition to metabolic effects, liraglutide has been studied for its effects on redox status and inflammation. It is also especially effective at reversing disease progression when used with statins [68]. Therefore, investigating drug interactions in this manner may provide the impetus for the development and exploration of effective therapeutic combinations aimed at improving treatment profiles in obesity-related metabolic diseases.

In the current study, a rat model of obesity and hyperlipidemia was successfully developed by feeding an HFD to rats for 12 weeks. Interestingly, treatment with atorvastatin did not lead to a reduction in weight gain, which supports previous observations that statins have little to no direct effect on body weight regulation through their primary function of lipid lowering via HMG-CoA reductase inhibition [69,70]. Several studies show that atorvastatin does not halt weight gain or could be associated with slight weight increases due to lower circulating leptin levels, which are the principal regulatory hormonal signal for plant-based satiety and food intake. Its principal action is lipid-lowering mediated by HMG-CoA reductase inhibition, with no primary effect on body weight [71]. In contrast, liraglutide treatment elicited a significant reduction in body weight, attributable to its pharmacological activity as a glucagon-like peptide-1 (GLP-1) receptor agonist. This effect is mediated through central nervous system pathways that enhance satiety and suppress appetite, coupled with peripheral actions including delayed gastric emptying and, in certain preclinical and clinical models, increased energy expenditure [72]. The most noticeable body weight decrease was achieved when atorvastatin was combined with liraglutide, and they reduced body weight statistically significantly versus either treatment alone. To achieve a pragmatic conclusion, atorvastatin may be treating dyslipidemia while liraglutide is efficiently counteracting weight gain acquired from the high-fat diet, suggesting a real therapeutic benefit of combining them in treatment for obesity.

It is known that the nuclear factor erythroid 2 2-related factor 2 (Nrf2)/heme oxygenase-1 (HO-1) signaling pathway represents a major cellular source of defense from oxidative and inflammatory insult. It has been suggested that dysregulation of this pathway may mediate obesity-induced metabolic dysfunction; thus, Nrf2/HO-1 emerges as a potentially valuable therapeutic target [21,73]. Though both atorvastatin and liraglutide have been noted to influence Nrf2/HO-1 activity, very little is known about the combined effects of these two drugs in the context of diet-induced obesity and fatty liver disease. Given these factors, exploring the combined effects of atorvastatin and liraglutide on Nrf2/HO-1 signaling in liver and adipose tissue provides context about potential complementary or synergistic effects. Our findings revealed that rats on a high-fat diet (HFD) exhibit greater Nrf2 and HO-1 mRNA expression than the normal diet (ND) group. This observation could be due to a compensatory defense response to oxidative stress, which is further evident by the treatment of either ATV or liraglutide, leading to upregulation of HO-1 mRNA expression or Nrf2 mRNA expression above levels observed in the HFD group. Taken together, these results suggest that both ATV and liraglutide amplify the effects of endogenous antioxidant changes regulated by the Nrf2/HO-1 pathway, particularly the combination dosage of ATV and liraglutide that led to the greatest overall change, which exhibited significant differences in Nrf2 and HO-1 expression compared to the other groups. By comparison, Di Veroli et al. reported changes in Nrf2 nuclear localization and HO-1 expression, providing evidence that the Nrf2/HO-1 pathway may participate in metabolic stress associated with changes in nutrients [74]. Also, Zhang et al. indicated that antioxidant treatments or drug interventions can facilitate Nrf2/HO-1 activation and enhance redox homeostasis and inflammation in HFD and obesity models. Although there may be limited actual studies that combine atorvastatin and liraglutide at the mRNA level for Nrf2/HO-1 with rats, Zhang et al.’s findings lend support to the mechanistic rationale for their respective potentiation [75]. This combination additive benefit further emphasizes the notion of potentially complementary mechanisms of the two drugs, whereby liraglutide may further enhance atorvastatin’s antioxidant and cytoprotective function. Overall, the results are consistent with the hypothesis that co-treatment provides a more effective modulatory effect on redox homeostasis and inflammation via Nrf2/HO-1 pathway activation, the principal mediator of the improved metabolic and hepatic outcomes.

Consistent with the established role of oxidative stress in obesity-associated metabolic dysfunction, rats fed a high-fat diet (HFD) exhibited significantly reduced levels of key antioxidant defenses—superoxide dismutase (SOD) and glutathione (GSH)—in both serum and hepatic tissue. This antioxidant depletion was accompanied by a marked elevation in malondialdehyde (MDA), a well-validated biomarker of lipid peroxidation and oxidative stress. These findings indicate that HFD feeding creates a pro-oxidant environment, systemically and within the liver. Treatment with atorvastatin or liraglutide partially reversed the pro-oxidant state, as increased SOD and GSH with lower MDA levels were observed, compared to the HFD group of rats. In this regard, both atorvastatin and liraglutide should have the ability to reduce oxidative stress effects significantly. Importantly, the combination of atorvastatin and liraglutide provided the most profound effect on reducing MDA and restoring both SOD and GSH levels to those similar to the ND. Consistent with our findings, others have shown that HFD feeding in rats increases oxidative stress by increasing MDA, and subsequently decreases GSH and activities of antioxidant enzymes, such as SOD, in the liver and other tissues [76,77,78]. Our findings indicate a synergistic effect between the drugs toward restoring the reduced oxidative stress response. This is likely because of the many secondary effects of these drugs acting on multiple targets, resulting in systemic cellular protection against oxidative stress.

Considering the lipid profile assessment is fundamental as a key indicator of metabolic health, given that dyslipidemia is the metabolic signature of obesity, it is a significant contributor to cardiovascular complications [79]. Herein, we indicated that HFD-fed rats showed clear dyslipidemia, as observed in the stark increases in serum triglycerides, total cholesterol, and LDL, along with decreased HDL between groups compared to normal controls. We also showed a significant improvement in lipid parameters in rats treated with liraglutide, potentially improving lipid metabolism via improved insulin sensitivity to control hepatic lipid turnover. By comparison, we showed substantially greater improvements in the lipid parameters exhibited by atorvastatin; this is not surprising given that atorvastatin is a potent inhibitor of cholesterol biosynthesis via HMG-CoA reductase inhibition [80,81]. Within the included studies, both atorvastatin and liraglutide showed restorations of lipid levels nearly back to normal, suggesting potential synergistic therapeutic effects, supporting our results [47,82]. Together, these findings have clear clinical merit in correcting lipid profile abnormalities with pharmacological intervention approaches centered on obesity related dyslipidemia.

According to adiponectin and leptin, our results are in line with the known pattern of HFD-induced metabolic dysfunction and support a synergistic benefit of combined atorvastatin/liraglutide on adipokine balance and insulin sensitivity [47,83]. Leptin is elevated, and adiponectin is reduced in obesity and high-fat diets (HDF), both of which are indicators of an inability of adipose to work properly, resulting in insulin resistance and low-grade inflammation. The relatively low level of adiponectin (1.30 ng/mL) and the comparatively high level of leptin (12.88 ng/mL) in the HFD groups indicate the typical pattern of dysregulation of adipokines and leptin resistance associated with obesity induced by a HDF [84,85]. In addition, the liraglutide group shows an increase in adiponectin level (2.78 ng/mL) as well as a significant decrease in leptin (5.93 ng/mL), indicating a more favorable adiponectin/leptin ratio and suggesting better restoration of insulin sensitivity than with atorvastatin alone [84]. Atorvastatin/liraglutide in combination has been shown to have both additive vasculoprotective and metabolic effects when given together compared to either medication alone.

In addition, adiponectin has been shown to have an insulin-sensitizing effect, an anti-inflammatory effect, and to protect the liver, with a direct association between low levels of adiponectin and the development of insulin resistance due to a HFD and/or accumulation of ectopic lipid in other areas. However, significantly elevated leptin levels with an inability to respond to leptin result in continued weight gain and metabolic disturbance [85,86]. Thus, the progressive improvement from our results indicates stepwise correction of adipose tissue dysfunction, with the combination regimen most effectively restoring the adiponectin-leptin axis and, by implication, metabolic homeostasis.

Chronic low-grade inflammation is a well-established hallmark of obesity and high-fat diet (HFD)-induced metabolic dysregulation. Pro-inflammatory cytokines—particularly interleukin-1β (IL-1β) and tumor necrosis factor-α (TNF-α)—serve as central mediators in the pathogenesis of insulin resistance, hepatic steatosis, and the broader spectrum of metabolic syndrome, linking adipose tissue dysfunction to systemic metabolic impairment [83,84]. Consistent with this, our data showed a significant increase in hepatic and serum IL-1β and TNF-α levels in the HFD cohort compared to the ND, confirming an inflammatory state. Treatment with atorvastatin or liraglutide alone significantly reduced the levels of both cytokines, consistent with their known anti-inflammatory effects. Atorvastatin has been shown to reduce levels of IL-1β and TNF-α by reducing inflammatory responses, in part, through its modulation of NF-κB signaling and other non-lipid-lowering pathways. This has been shown in vivo in animal models where atorvastatin reduced pro-inflammatory cytokines in serum and tissue and contributed in part to its anti-inflammatory and plaque-stabilizing effects [87]. Likewise, liraglutide produces anti-inflammatory effects by reducing adipose tissue inflammatory state, increasing insulin sensitivity, and inhibiting NF-κB activation, which reduces IL-1β and TNF-α production [88]. The anti-inflammatory capability of liraglutide has been shown in many models, including liver injury and HFD [72]. Notably, both drugs used resulted in greater suppression of IL-1β and TNF-α, with the levels of these two pro-inflammatory cytokines also approaching normal levels. This indicates that dual targeting lipid metabolism and inflammatory signaling activation, unlike targeting either lipid metabolism or inflammatory signaling systems alone, can result in a synergistic mechanism of action. Taken together, these data indicate the additive benefits of combining lipid-lowering agents (statins) with GLP-1 receptor agonists to combat obesity-related inflammation using a mechanistic combination of targeting lipolysis and inflammatory signaling.

Dysregulated glucose homeostasis is a significant metabolic consequence of high-fat diet intake, typically characterized by increased hyperglycemia and HbA1c levels, both of which are powerful predictors of insulin resistance and type 2 diabetes risk [89]. In our study, we observed that HFD rats had a significant elevation in FBG and HbA1c levels when compared to ND, indicating impaired glycemic control. Based on previous reports relating statin therapy to altered glucose metabolism and increased risk of developing new-onset diabetes in at-risk individuals [90]. Atorvastatin treatment alone caused a small but insignificant increase in FBG and HbA1c. On the other hand, liraglutide monotherapy resulted in a significant reduction in FBG and HbA1c, which is consistent with its glucose-lowering effects through its mechanisms of increasing insulin secretion, delaying gastric emptying, and reducing appetite suppression while demonstrating its glucose-lowering effects in diabetic and HFD models [72]. The best results from our study were from the atorvastatin/liraglutide treatment, which returned glucose and HbA1c levels to those of ND. This implies that liraglutide may antagonize the adverse glycemic effects of atorvastatin while promoting the therapeutic effects of atorvastatin, providing a strong rationale for combination treatment for a patient population with concurrent dyslipidemia.

Also, our data suggest that HFD feeding significantly induced hepatic COX-2 expression as an indication of enhanced hepatic inflammation. These results fit with previous studies reporting COX-2 upregulation under metabolic stress, and that COX-2 contributes to steatosis and hepatic inflammation progression [91]. The atorvastatin treatment decreased COX-2 expression significantly, likely due to both lipid-lowering and pleiotropic anti-inflammatory effects separate from lipid-lowering. As outlined by Peng et al., atorvastatin treatment could reduce the inflammatory response and COX-2 expression in macrophage and liver models [85]. Liraglutide treatment induced a more significant reduction in COX-2, potentially through its ability to modulate inflammatory signaling and improve metabolic state, as it decreases COX-2 and TNF-α in two separate hepatic and macrophage models, all indicating its anti-inflammatory and hepatoprotective effects [72]. Treatment with both atorvastatin and liraglutide had the greatest impact on COX-2. The additive effect of the two treatments suggests the therapeutic benefit of treatment with statins and GLP-1 receptor agonists to treat obesity-induced hepatic inflammation.

In our study, the administration of an HFD significantly increased LC3 expression while concurrently decreasing p62 expression in liver tissue. This outcome suggests an activation of autophagy in addition to impaired degradation. These results are in agreement with previous reports relating obesity-associated oxidative and inflammatory stress, dysregulation of autophagic flux, and impaired degradation pathways [92,93]. Treatment with atorvastatin or liraglutide partially reversed these changes based on decreased LC3 and restored p62 expression, indicating that both drugs can influence autophagy. Interestingly, combined atorvastatin and liraglutide therapy had a more profound effect than either drug alone and restored both LC3 and p62 expression towards control values. It was reported in other publications that oxidative stress and inflammation associated with obesity dysregulate autophagy and mediate impaired degradation pathways and changed p62 and LC3 expression, and this supports the potential connections among metabolic stress and disruption of autophagy. Korovila et al. showed that a high-fat diet disrupted autophagy in liver tissue, with altered levels of autophagy proteins such as LC3, as well as excess and depletion of p62, as autophagic flux was disrupted [92]. Liraglutide was indicated to be able to enhance autophagy through AMPK and associated signaling; reduce inflammation and oxidative stress; and, overall, improve liver function. Atorvastatin with liraglutide indicated additive effects as both would result in benefits of improvements in autophagic balance and hepatic function. It may be that the two pathways of atorvastatin and liraglutide would work synergistically, as atorvastatin would reduce the burden of fatty accumulation and oxidative stress, while liraglutide would improve insulin sensitivity and inflammatory response, thus enhancing each other’s properties to restore autophagy. All evidence suggests that combined therapy targets multiple pathways at restoring an autophagic balance that may have been impacted by combinations of higher fat feeding, thus severely contributing to hepatic dysfunction.

Finally, our histological findings support the biochemical findings in this study and provide morphological evidence of hepatic injury. The HFD group had predominating pathological features of hepatocyte injury, which were the presence of hepatocyte ballooning, Mallory Denk body formation, apoptotic changes, as well as increased collagen deposition—thus confirming the HFD group was suffering from steatohepatitis and early fibrotic changes. These impairments were similar to the prior entities that were associated with chronic feeding of an HFD. We have shown that treatment with either atorvastatin, liraglutide, or both decreased some of the pathological features of hepatocyte injury, as indicated by reduced ballooning of hepatocytes, fewer Mallory–Denk bodies, and less collagen deposition. Of interest was the combination group, which came close to demonstrating typical histological characteristics. In particular, Elsiad et al., authors of a previous article, showed that liraglutide treatment reduced atorvastatin-induced liver injury in a rat model. The study reported improvements in liver function tests, as well as substantial histological findings that included decreased hepatocyte necrosis, inflammation, and fibrosis. The research demonstrated that liraglutide contains antioxidant and anti-inflammatory properties, activates autophagy pathways, and reduces apoptosis, which leads to hepatoprotection. Furthermore, the hepatoprotective benefits of statins and GLP-1 receptor agonists like liraglutide in MAFLD and Metabolic Dysfunction-Associated Steatohepatitis (MASH) led to fibrosis and inflammation improvement, supporting the synergistic benefits of these drugs [68]. Thus, the structural hepatoprotection indicated that the benefits of atorvastatin and liraglutide were not only due to metabolic benefits but also via a structural reduction in the progression of steatohepatitis and fibrogenesis. Moreover, taken together, the histological evidence of the study affirms and endorses the biochemical evidence, corroborating the possibility of synergy benefits of these drugs in improving diet-induced hepatic injury.

Molecular docking simulations demonstrated that atorvastatin binds favorably to both Nrf2 (PDB ID: 5CGJ) and HO-1 (PDB ID: 1N3U), with binding energies of −7.98 kcal/mol and −7.22 kcal/mol, respectively. The interaction with Nrf2 was primarily stabilized by hydrogen bonds with Ser363, Ile416, and Ser555, as well as a π–π-cation interaction with Arg415, suggesting a potential modulatory effect on the Nrf2–ARE signaling pathway. In the HO-1 complex, atorvastatin formed hydrogen bonds with Tyr134, Lys179, and Arg183, complemented by π–π, amide–π, and π–sulfur interactions with His25, Ser142, and Met34, respectively, reflecting a diverse network of stabilizing contacts within the enzyme’s active site.

The interaction profile of atorvastatin is consistent with its amphiphilic nature, where polar groups facilitate hydrogen bonding and aromatic moieties promote π-mediated interactions. The stronger predicted binding toward Nrf2 suggests a direct influence on antioxidant and cytoprotective signaling. At the same time, the complex interaction network with HO-1 implies a secondary role in modulating heme degradation and oxidative stress regulation. These in silico findings correlate with in vivo observations in high-fat diet (HFD)-fed rats. Atorvastatin treatment partially improved metabolic parameters, including body weight, serum cholesterol, and hepatic COX-2 expression. Liraglutide showed enhanced efficacy, and combination therapy produced the most significant effect, normalizing body weight, glucose, lipid profiles, and COX-2 levels while reactivating the Nrf2/HO-1 pathway, as evidenced by increased HO-1 expression and restoration of p62 levels. Collectively, these data suggest that atorvastatin and liraglutide may synergistically target Nrf2 and HO-1 to enhance antioxidant and anti-inflammatory responses in hepatic and adipose tissues.

Complementing the small-molecule docking results, AlphaFold-Multimer modeling of liraglutide with Nrf2 and HO-1 provided high-confidence structural predictions that offer mechanistic insights into peptide-mediated modulation of these proteins. The top-ranked Nrf2–liraglutide model exhibited an average predicted Local Distance Difference Test (pLDDT) of 82.2 and an interface TM-score (ipTM) of 0.362, indicating reliable backbone and side-chain modeling and plausible interactions at regulatory and DNA-binding regions (Figure 15). Similarly, the best HO-1 model showed an average pLDDT of 81.4 and an ipTM of 0.619, suggesting stable engagement of the peptide at the enzyme’s catalytic and regulatory interface (Figure 16). Visual inspection highlighted stabilizing contacts, including hydrogen bonds and polar interactions, supporting both targets’ direct peptide–protein engagement.

Although AlphaFold-Multimer does not provide quantitative binding energies, the structural metrics indicate that liraglutide adopts conformations compatible with the functional regions of Nrf2 and HO-1. Binding to Nrf2 may stabilize or modulate conformational states necessary for nuclear translocation and transcriptional activation of antioxidant genes. In contrast, interaction with HO-1 may enhance enzymatic activity or structural stability, facilitating heme degradation and cytoprotective responses. These predictions align with the in vivo findings, in which liraglutide, particularly in combination with atorvastatin, restored disturbed antioxidant markers (SOD, GSH, p62) and reduced inflammatory mediators, reactivating the Nrf2/HO-1 signaling pathway.

Overall, the integration of docking simulations and AlphaFold-Multimer modeling provides a mechanistic framework in which atorvastatin and liraglutide modulate oxidative stress and inflammation through complementary mechanisms. Atorvastatin engages key residues within Nrf2 and HO-1 to modulate transcriptional and enzymatic activity, while liraglutide stabilizes structurally relevant interfaces, enhancing cytoprotective and antioxidant signaling. These findings underscore the therapeutic potential of a combined atorvastatin–liraglutide strategy to ameliorate obesity-related metabolic dysfunction through coordinated modulation of the Nrf2/HO-1 signaling axis.

## 5. Conclusions

Treatment with atorvastatin or liraglutide alleviated some of the changes observed, with both atorvastatin and liraglutide decreasing blood lipids, and liraglutide uniquely reducing blood glucose and body weight. Both atorvastatin and liraglutide increased Nrf2/HO-1 expression and increased activity of antioxidant enzymes to dramatically increase levels of oxidative stress and inflammatory cytokines, which may be associated with changes noted histologically. Furthermore, the combination of atorvastatin and liraglutide caused a greater therapeutic response than either atorvastatin or liraglutide alone; in fact, there is evidence of a synergistic effect with this combination.

As for the in-silico confirmation, molecular docking simulations of atorvastatin demonstrated favorable binding to both Nrf2 and HO-1, providing a mechanistic rationale for its ability to modulate these oxidative stress regulators. Furthermore, AlphaFold-Multimer modeling revealed that liraglutide engages structurally relevant interfaces on Nrf2 and HO-1, supporting its potential to stabilize or activate these proteins and enhance antioxidant and cytoprotective signaling.

Overall, our findings support modulation of the Nrf2/HO-1 signaling pathway as a potential therapeutic target for reversing hyperlipidemia and preventing mediators of inflammation and oxidative stress damage in hepatic tissue. The evidence of increased efficacy observed with the combined atorvastatin and liraglutide supports a potential novel understanding of the complementary effects of atorvastatin and liraglutide. It supports future studies to understand the potential advantage in metabolic disorder scenarios.

## Figures and Tables

**Table 1:** Primers used in this study.

Gene		Sequence	References
**LC3**	Forward	GGTCCAGTTGTGCCTTTATTGA	[51]
	Reverse	GTGTGTGGGTTGTGTACGTCG	
**p62**	Forward	CTAGGCATCGAGGTTGACATT	[51]
	Reverse	CTTGGCTGAGTACCACTCTTATC	
**GAPDH**	Forward	GTTACCAGGGCTGCCTTCTC	[51]
	Reverse	GATGGTGATGGGTTTCCCGT	
**COX-2**	Forward	CAGACAACATAAACTGCGCCTT	[52]
	Reverse	GATACACCTCTCCACCAATGACC	

**Table 2:** The effect of atorvastatin and liraglutide on body weight.

Animal Groups (*n* = 10)	Body Weight (After Week 1)	Body Weight (After Week 12)
ND	157 ± 4.5	260
HFD	155 ± 5.2	316 *
HFD/atorvastatin	156 ± 4.8	320 *
HFD/liraglutide	152 ± 6.3	240 **
HFD/Atorvastatin + Lira	155 ± 5.1	224 ***

Significance: * *p* < 0.05, ** *p* < 0.01, *** *p* < 0.001, (one-way ANOVA followed by Tukey’s post hoc test). Red symbols indicate significance compared to the ND group; blue symbols indicate significance compared to the HFD group; black symbols indicate significance compared to HFD treated with ATV or Liraglutide. Data is expressed as mean and SD (standard deviation), number of animals = 6 per group. ATV: atorvastatin, lira: liraglutide.

**Table 3:** Molecular docking results of atorvastatin with Nrf2 (PDB ID: 5CGJ) and HO-1 (PDB ID: 1N3U). Binding energies (kcal/mol) and key amino acid residues involved in hydrogen bonding and noncovalent interactions are listed.

Target Protein	Binding Energy (kcal/mol)	Residue	Type of Interaction
Nrf2, PDB ID: 5CGJ	−7.98	Ser363	H-bond
		Ile416	H-bond
		Ser555	H-bond
		Arg415	π-cation
HO-1, PDB ID: 1N3U	−7.22	Tyr134	H-bond
		Lys179	H-bond
		Arg183	H-bond
		His25	π-π stacking
		Ser142	Amide-π stacking
		Met34	π-Sulfur

## Data Availability

The data that support the findings of this study are available from the first and corresponding authors upon request.

## References

[1] Chew N.W.S., Ng C.H., Tan D.J.H., Kong G., Lin C., Chin Y.H., Lim W.H., Huang D.Q., Quek J., Fu C.E. (2023). The Global Burden of Metabolic Disease: Data from 2000 to 2019. Cell Metab..

[2] Chen R., Safiri S., Behzadifar M., Kong J.D., Zguira M.S., Bragazzi N.L., Zhong W., Zhang W. (2022). Health Effects of Metabolic Risks in the United States from 1990 to 2019. Front. Public Health.

[3] Mohamed D., Ezzat S., Elayat W., El-Kharashi O., El-Kareem H., Nahas H., Abdel-Wahab B., Alshawwa S., Saleh A., Helmy Y. (2022). Hepatoprotective Role of Carvedilol against Ischemic Hepatitis Associated with Acute Heart Failure via Targeting miRNA-17 and Mitochondrial Dynamics-Related Proteins: An In Vivo and In Silico Study. Pharmaceuticals.

[4] Islam M.S., Wei P., Suzauddula M., Nime I., Feroz F., Acharjee M., Pan F. (2024). The Interplay of Factors in Metabolic Syndrome: Understanding Its Roots and Complexity. Mol. Med..

[5] Roy P.K., Islam J., Lalhlenmawia H. (2023). Prospects of Potential Adipokines as Therapeutic Agents in Obesity-Linked Atherogenic Dyslipidemia and Insulin Resistance. Egypt. Heart J..

[6] Kumar Khemka V., Banerjee A. (2017). Metabolic Risk Factors in Obesity and Diabetes Mellitus: Implications in the Pathogenesis and Therapy. Integr Obes. Diabetes.

[7] Marseglia L., Manti S., D’Angelo G., Nicotera A., Parisi E., Di Rosa G., Gitto E., Arrigo T. (2015). Oxidative Stress in Obesity: A Critical Component in Human Diseases. Int. J. Mol. Sci..

[8] Fonseca-Alaniz M.H., Takada J., Alonso-Vale M.I.C., Lima F.B. (2007). Adipose Tissue as an Endocrine Organ: From Theory to Practice. J. Pediatr. (Rio J.).

[9] Wang B., Trayhurn P. (2006). Acute and Prolonged Effects of TNF-α on the Expression and Secretion of Inflammation-Related Adipokines by Human Adipocytes Differentiated in Culture. Pflüg. Arch..

[10] Neira G., Gómez-Ambrosi J., Cienfuegos J.A., Ramírez B., Becerril S., Rodríguez A., Burrell M.A., Baixauli J., Mentxaka A., Casado M. (2025). Increased Expression of IL-1β in Adipose Tissue in Obesity Influences the Development of Colon Cancer by Promoting Inflammation. J. Physiol. Biochem..

[11] Martemucci G., Fracchiolla G., Muraglia M., Tardugno R., Dibenedetto R.S., D’Alessandro A.G. (2023). Metabolic Syndrome: A Narrative Review from the Oxidative Stress to the Management of Related Diseases. Antioxidants.

[12] Tauil R.B., Golono P.T., de Lima E.P., de Alvares Goulart R., Guiguer E.L., Bechara M.D., Nicolau C.C.T., Yanaguizawa Junior J.L., Fiorini A.M.R., Méndez-Sánchez N. (2024). Metabolic-Associated Fatty Liver Disease: The Influence of Oxidative Stress, Inflammation, Mitochondrial Dysfunctions, and the Role of Polyphenols. Pharmaceuticals.

[13] Park J.S., Rustamov N., Roh Y.S. (2023). The Roles of NFR2-Regulated Oxidative Stress and Mitochondrial Quality Control in Chronic Liver Diseases. Antioxidants.

[14] Allameh A., Niayesh-mehr R., Aliarab A., Sebastiani G., Pantopoulos K. (2023). Oxidative Stress in Liver Pathophysiology and Disease. Antioxidants.

[15] Loboda A., Damulewicz M., Pyza E., Jozkowicz A., Dulak J. (2016). Role of Nrf2/HO-1 System in Development, Oxidative Stress Response and Diseases: An Evolutionarily Conserved Mechanism. Cell. Mol. Life Sci..

[16] Bukke V.N., Moola A., Serviddio G., Vendemiale G., Bellanti F. (2022). Nuclear Factor Erythroid 2-Related Factor 2-Mediated Signaling and Metabolic Associated Fatty Liver Disease. World J. Gastroenterol..

[17] Hasan S.K., Jayakumar S., Espina Barroso E., Jha A., Catalano G., Sandur S.K., Noguera N.I. (2025). Molecular Targets of Oxidative Stress: Focus on Nuclear Factor Erythroid 2–Related Factor 2 Function in Leukemia and Other Cancers. Cells.

[18] Liu S., Pi J., Zhang Q. (2022). Signal Amplification in the KEAP1-NRF2-ARE Antioxidant Response Pathway. Redox Biol..

[19] Saha S., Buttari B., Panieri E., Profumo E., Saso L. (2020). An Overview of Nrf2 Signaling Pathway and Its Role in Inflammation. Molecules.

[20] Xia Y., Zhai X., Qiu Y., Lu X., Jiao Y. (2022). The Nrf2 in Obesity: A Friend or Foe?. Antioxidants.

[21] Li N., Hao L., Li S., Deng J., Yu F. (2024). The NRF-2/HO-1 Signaling Pathway: A Promising Therapeutic Target for Metabolic Dysfunction-Associated Steatotic Liver Disease. J. Inflamm. Res..

[22] Zhang X., Ding M., Zhu P., Huang H., Zhuang Q., Shen J., Cai Y., Zhao M., He Q. (2019). New Insights into the Nrf-2/HO-1 Signaling Axis and Its Application in Pediatric Respiratory Diseases. Oxid. Med. Cell. Longev..

[23] Solano-Urrusquieta A., Morales-González J.A., Castro-Narro G.E., Cerda-Reyes E., Flores-Rangel P.D., Fierros-Oceguera R. (2020). NRF-2 and Nonalcoholic Fatty Liver Disease. Ann. Hepatol..

[24] Wang L., He C. (2022). Nrf2-Mediated Anti-Inflammatory Polarization of Macrophages as Therapeutic Targets for Osteoarthritis. Front. Immunol..

[25] Galicia-moreno M., Lucano-landeros S., Monroy-ramirez H.C., Silva-gomez J., Gutierrez-cuevas J., Santos A., Armendariz-borunda J. (2020). Roles of NRF2 in Liver Diseases: Molecular, Pharmacological, and Epigenetic Aspects. Antioxidants.

[26] Chambel S.S., Santos-Gonçalves A., Duarte T.L. (2015). The Dual Role of Nrf2 in Nonalcoholic Fatty Liver Disease: Regulation of Antioxidant Defenses and Hepatic Lipid Metabolism. BioMed Res. Int..

[27] Bathish B., Robertson H., Dillon J.F., Dinkova-Kostova A.T., Hayes J.D. (2022). Nonalcoholic Steatohepatitis and Mechanisms by Which It Is Ameliorated by Activation of the CNC-bZIP Transcription Factor Nrf2. Free Radic. Biol. Med..

[28] Shin S., Wakabayashi N., Misra V., Biswal S., Lee G.H., Agoston E.S., Yamamoto M., Kensler T.W. (2007). NRF2 Modulates Aryl Hydrocarbon Receptor Signaling: Influence on Adipogenesis. Mol. Cell. Biol..

[29] Xu J., Donepudi A.C., More V.R., Kulkarni S.R., Li L., Guo L., Yan B., Chatterjee T., Weintraub N., Slitt A.L. (2015). Deficiency in Nrf2 Transcription Factor Decreases Adipose Tissue Mass and Hepatic Lipid Accumulation in Leptin-Deficient Mice. Obesity.

[30] Tonelli C., Chio I.I.C., Tuveson D.A. (2018). Transcriptional Regulation by Nrf2. Antioxid. Redox Signal..

[31] Wang Y., Fu X., Zeng L., Hu Y., Gao R., Xian S., Liao S., Huang J., Yang Y., Liu J. (2024). Activation of Nrf2/HO-1 Signaling Pathway Exacerbates Cholestatic Liver Injury. Commun. Biol..

[32] Bellezza I., Giambanco I., Minelli A., Donato R. (2018). Nrf2-Keap1 Signaling in Oxidative and Reductive Stress. Biochim. Biophys. Acta Mol. Cell Res..

[33] Balasubramanian R., Maideen N.M.P. (2021). HMG-CoA Reductase Inhibitors (Statins) and Their Drug Interactions Involving CYP Enzymes, P-Glycoprotein and OATP Transporters-An Overview. Curr. Drug Metab..

[34] Khatiwada N., Hong Z. (2024). Potential Benefits and Risks Associated with the Use of Statins. Pharmaceutics.

[35] Khalifeh M., Penson P.E., Banach M., Sahebkar A. (2021). Statins as Anti-Pyroptotic Agents. Arch. Med. Sci..

[36] Luedde T., Schwabe R.F. (2011). NF-κB in the Liver-Linking Injury, Fibrosis and Hepatocellular Carcinoma. Nat. Rev. Gastroenterol. Hepatol..

[37] Mansouri A., Reiner Ž., Ruscica M., Tedeschi-Reiner E., Radbakhsh S., Ekta M.B., Sahebkar A. (2022). Antioxidant Effects of Statins by Modulating Nrf2 and Nrf2/HO-1 Signaling in Different Diseases. J. Clin. Med..

[38] Jaikumkao K., Pongchaidecha A., Thongnak L.O., Wanchai K., Arjinajarn P., Chatsudthipong V., Chattipakorn N., Lungkaphin A. (2016). Amelioration of Renal Inflammation, Endoplasmic Reticulum Stress and Apoptosis Underlies the Protective Effect of Low Dosage of Atorvastatin in Gentamicin-Induced Nephrotoxicity. PLoS ONE.

[39] Marková I., Malínská H., Hüttl M., Miklánková D., Oliyarnyk O., Poruba M., Rácová Z., Kazdová L., Večeřa R. (2021). The Combination of Atorvastatin With Silymarin Enhances Hypolipidemic, Antioxidant and Anti-Inflammatory Effects in a Rat Model of Metabolic Syndrome. Physiol. Res..

[40] Prajapati S.K., Garabadu D., Krishnamurthy S. (2017). Coenzyme Q10 Prevents Mitochondrial Dysfunction and Facilitates Pharmacological Activity of Atorvastatin in 6-OHDA Induced Dopaminergic Toxicity in Rats. Neurotox. Res..

[41] Tangelloju S., Little B.B., Esterhay R.J., Brock G., LaJoie S. (2020). Statins Are Associated with New Onset Type 2 Diabetes Mellitus (T2DM) in Medicare Patients ≥65 Years. Diabetes Metab. Res. Rev..

[42] Al Qassab M., Mneimneh M., Jradi A., Derbas B., Dabboussi D., Khoury Baini J., Katrib N., Chaarani N., Attieh P., Kanaan A. (2025). The Expanding Role of GLP-1 Receptor Agonists: Advancing Clinical Outcomes in Metabolic and Mental Health. Curr. Issues Mol. Biol..

[43] Movahednasab M., Dianat-Moghadam H., Khodadad S., Nedaeinia R., Safabakhsh S., Ferns G., Salehi R. (2025). GLP-1-Based Therapies for Type 2 Diabetes: From Single, Dual and Triple Agonists to Endogenous GLP-1 Production and L-Cell Differentiation. Diabetol. Metab. Syndr..

[44] Piccirillo F., Mastroberardino S., Nusca A., Frau L., Guarino L., Napoli N., Ussia G.P., Grigioni F. (2023). Novel Antidiabetic Agents and Their Effects on Lipid Profile: A Single Shot for Several Cardiovascular Targets. Int. J. Mol. Sci..

[45] Han X., Ding C., Zhang G., Pan R., Liu Y., Huang N., Hou N., Han F., Xu W., Sun X. (2020). Liraglutide Ameliorates Obesity-Related Nonalcoholic Fatty Liver Disease by Regulating Sestrin2-Mediated Nrf2/HO-1 Pathway. Biochem. Biophys. Res. Commun..

[46] Sedky A.A., Magdy Y. (2021). Reduction in TNF Alpha and Oxidative Stress by Liraglutide: Impact on Ketamine-Induced Cognitive Dysfunction and Hyperlocomotion in Rats. Life Sci..

[47] El Medany A.M.H., Hammadi S.H.M., Khalifa H.M., Ghazala R.A., Zakaria Mohammed H.S. (2022). The Vascular Impact of Dapagliflozin, Liraglutide, and Atorvastatin Alone or in Combinations in Type 2 Diabetic Rat Model. Fundam. Clin. Pharmacol..

[48] Kaur N., Kishore L., Singh R. (2018). *Dillenia indica* L. Attenuates Diabetic Nephropathy via Inhibition of Advanced Glycation End Products Accumulation in STZ-Nicotinamide Induced Diabetic Rats. J. Tradit. Complement. Med..

[49] Inada M., Oishi M., Nishikawa M., Kurata S., Imura H. (1980). Clinical Evaluation of Measuring Glycosylated Hemoglobin Levels for Assessing the Long-Term Blood Glucose Control in Diabetics. Endocrinol. Jpn..

[50] Deshpande K.C., Kulkarni M.M., Rajput D.V. (2023). Evaluation of Glutathione Peroxidase in the Blood and Tumor Tissue of Oral Squamous Cell Carcinoma Patients. J. Oral Maxillofac. Pathol..

[51] Tarawan V.M., Gunadi J.W., Setiawan, Lesmana R., Goenawan H., Meilina D.E., Sipayung J.A., Wargasetia T.L., Widowati W., Limyati Y. (2019). Alteration of Autophagy Gene Expression by Different Intensity of Exercise in Gastrocnemius and Soleus Muscles of Wistar Rats. J. Sports Sci. Med..

[52] Ojeaburu S.I., Oriakhi K. (2021). Hepatoprotective, Antioxidant and, Anti-Inflammatory Potentials of Gallic Acid in Carbon Tetrachloride-Induced Hepatic Damage in Wistar Rats. Toxicol. Rep..

[53] Hanwell M.D., Curtis D.E., Lonie D.C., Vandermeersch T., Zurek E., Hutchison G.R. (2012). Avogadro: An Advanced Semantic Chemical Editor, Visualization, and Analysis Platform. J. Cheminform..

[54] Gasteiger J., Marsili M. (1978). A New Model for Calculating Atomic Charges in Molecules. Tetrahedron Lett..

[55] Morris M.G., Goodsell D.S., Halliday R.S., Huey R., Hart W.E., Belew R.K., Olson A.J. (1998). Automated Docking Using a Lamarckian Genetic Algorithm and an Empirical Binding Free Energy Function. J. Comput. Chem..

[56] Kim G., Lee S., Levy Karin E., Kim H., Moriwaki Y., Ovchinnikov S., Steinegger M., Mirdita M. (2025). Easy and Accurate Protein Structure Prediction Using ColabFold. Nat. Protoc..

[57] Meng E.C., Goddard T.D., Pettersen E.F., Couch G.S., Pearson Z.J., Morris J.H., Ferrin T.E. (2023). UCSF ChimeraX: Tools for Structure Building and Analysis. Protein Sci..

[58] Alyousef A.M., Mekawy D.Z., Bashumeel Y.Y., Mohamed S.M., Almigbal T.H., Batais M.A., Alrasheed A.A. (2025). The Prevalence of Metabolic Syndrome in Patients with Non-Alcoholic Fatty Liver Disease in Primary Care Clinics at King Saud University Medical City, Riyadh, Saudi Arabia. Front. Endocrinol..

[59] Bhargava B., Rao P.N., Kulkarni A.V., Vishnubhotla R., Pramod N., Anitha C.T., Mahadev K. (2025). Prevalence of Metabolic Dysfunction-Associated Fatty Liver Disease among Information Technology Employees in India. Sci. Rep..

[60] Mitrovic B., Gluvic Z.M., Obradovic M., Radunovic M., Rizzo M., Banach M., Isenovic E.R. (2023). Non-Alcoholic Fatty Liver Disease, Metabolic Syndrome, and Type 2 Diabetes Mellitus: Where Do We Stand Today?. Arch. Med. Sci..

[61] Mohamed D.I., Abo Nahas H.H., Elshaer A.M., El-Waseef D.A., El-Kharashi O.A., Mohamed S.M., Sabry Y.G., Almaimani R.A., Almasmoum H.A., Altamimi A.S. (2023). Unveiling the Interplay between NSAID-Induced Dysbiosis and Autoimmune Liver Disease in Children: Insights into the Hidden Gateway to Autism Spectrum Disorders. Evidence from Ex Vivo, in Vivo, and Clinical Studies. Front. Cell. Neurosci..

[62] Bays H.E., Kirkpatrick C.F., Maki K.C., Toth P.P., Morgan R.T., Tondt J., Christensen S.M., Dixon D.L., Jacobson T.A. (2024). Obesity, Dyslipidemia, and Cardiovascular Disease: A Joint Expert Review from the Obesity Medicine Association and the National Lipid Association 2024. J. Clin. Lipidol..

[63] Alqahtani M.S., Alzibali K.F., Mahdi A.M.M., Alharbi O.M.A., Harbi R.H.A., Alkhaldi H.S.M., Alsayafi Z.A.A., Albisher F.H., Buqurayn M.H., Alharbi M.M. (2024). Lipid-Lowering Medications for Managing Dyslipidemia: A Narrative Review. Cureus.

[64] Reis F. (2017). Therapeutic Strategies Targeting Oxidative Stress to Improve Dyslipidemia and Left Ventricular Hypertrophy. Cardiol. Port. J. Cardiol. Off. J. Port. Soc. Cardiol..

[65] Mohamed D., El-Waseef D.A.E.-D.A., Nabih E., El-Kharashi O., El-Kareem H.A., Nahas H.A., Abdel-Wahab B., Helmy Y., Alshawwa S., Saied E. (2022). Acetylsalicylic Acid Suppresses Alcoholism-Induced Cognitive Impairment Associated with Atorvastatin Intake by Targeting Cerebral miRNA155 and NLRP3: In Vivo, and In Silico Study. Pharmaceutics.

[66] Spoiala E.L., Cinteza E., Vatasescu R., Vlaiculescu M.V., Moisa S.M. (2024). Statins—Beyond Their Use in Hypercholesterolemia: Focus on the Pediatric Population. Children.

[67] Koushki K., Shahbaz S.K., Mashayekhi K., Sadeghi M., Zayeri Z.D., Taba M.Y., Banach M., Al-Rasadi K., Johnston T.P., Sahebkar A. (2021). Anti-Inflammatory Action of Statins in Cardiovascular Disease: The Role of Inflammasome and Toll-Like Receptor Pathways. Clin. Rev. Allergy Immunol..

[68] Barb D., Portillo-Sanchez P., Cusi K. (2016). Pharmacological Management of Nonalcoholic Fatty Liver Disease. Metab.-Clin. Exp..

[69] Ness G.C., Chambers C.M., Lopez D. (1998). Atorvastatin Action Involves Diminished Recovery of Hepatic HBG-CoA Reductase Activity. J. Lipid Res..

[70] Patel K.K., Sehgal V.S., Kashfi K. (2022). Molecular Targets of Statins and Their Potential Side Effects: Not All the Glitter Is Gold. Eur. J. Pharmacol..

[71] Abbasi F., Lamendola C., Harris C.S., Harris V., Tsai M.S., Tripathi P., Abbas F., Reaven G.M., Reaven P.D., Snyder M.P. (2021). Statins Are Associated With Increased Insulin Resistance and Secretion. Arterioscler. Thromb. Vasc. Biol..

[72] Meurot C., Martin C., Sudre L., Breton J., Bougault C., Rattenbach R., Bismuth K., Jacques C., Berenbaum F. (2022). Liraglutide, a Glucagon-like Peptide 1 Receptor Agonist, Exerts Analgesic, Anti-Inflammatory and Anti-Degradative Actions in Osteoarthritis. Sci. Rep..

[73] Li S., Eguchi N., Lau H., Ichii H. (2020). The Role of the Nrf2 Signaling in Obesity and Insulin Resistance. Int. J. Mol. Sci..

[74] Di Veroli B., Bentanachs R., Roglans N., Alegret M., Giona L., Profumo E., Berry A., Saso L., Laguna J.C., Buttari B. (2024). Sex Differences Affect the NRF2 Signaling Pathway in the Early Phase of Liver Steatosis: A High-Fat-Diet-Fed Rat Model Supplemented with Liquid Fructose. Cells.

[75] Zhang L., Li Y., Sun D., Bai F. (2022). Protective Effect of Nimbolide against High Fat Diet-Induced Obesity in Rats via Nrf2/HO-1 Pathway. J. Oleo Sci..

[76] Auberval N., Dal S., Bietiger W., Pinget M., Jeandidier N., Maillard-Pedracini E., Schini-Kerth V., Sigrist S. (2014). Metabolic and Oxidative Stress Markers in Wistar Rats after 2 Months on a High-Fat Diet. Diabetol. Metab. Syndr..

[77] Noeman S.A., Hamooda H.E., Baalash A.A. (2011). Biochemical Study of Oxidative Stress Markers in the Liver, Kidney and Heart of High Fat Diet Induced Obesity in Rats. Diabetol. Metab. Syndr..

[78] Lasker S., Rahman M.M., Parvez F., Zamila M., Miah P., Nahar K., Kabir F., Sharmin S.B., Subhan N., Ahsan G.U. (2019). High-Fat Diet-Induced Metabolic Syndrome and Oxidative Stress in Obese Rats Are Ameliorated by Yogurt Supplementation. Sci. Rep..

[79] Albitar O., D’Souza C.M., Adeghate E.A. (2024). Effects of Lipoproteins on Metabolic Health. Nutrients.

[80] Shachaf C.M., Perez O.D., Youssef S., Fan A.C., Elchuri S., Goldstein M.J., Shirer A.E., Sharpe O., Chen J., Mitchell D.J. (2007). Inhibition of HMGcoA Reductase by Atorvastatin Prevents and Reverses MYC-Induced Lymphomagenesis. Blood.

[81] Zeng W., Deng H., Luo Y., Zhong S., Huang M., Tomlinson B. (2025). Advances in Statin Adverse Reactions and the Potential Mechanisms: A Systematic Review. J. Adv. Res..

[82] Xiang L., Wu H., Zhao Z., Wu T., Lv D., Wu P., Zheng Y., Huang Q., Xu T. (2025). Lipid-Lowering Effect of Combined Therapy with High-Intensity Statins and CETP Inhibitors: A Systematic Review and Meta-Analysis. Front. Endocrinol..

[83] Tarantino G., Savastano S., Colao A. (2010). Hepatic Steatosis, Low-Grade Chronic Inflammation and Hormone/Growth Factor/Adipokine Imbalance. World J. Gastroenterol..

[84] Zatterale F., Longo M., Naderi J., Raciti G.A., Desiderio A., Miele C., Beguinot F. (2020). Chronic Adipose Tissue Inflammation Linking Obesity to Insulin Resistance and Type 2 Diabetes. Front. Physiol..

[85] Peng S., Xu L.W., Che X.Y., Xiao Q.Q., Pu J., Shao Q., He B. (2018). Atorvastatin Inhibits Inflammatory Response, Attenuates Lipid Deposition, and Improves the Stability of Vulnerable Atherosclerotic Plaques by Modulating Autophagy. Front. Pharmacol..

[86] Mohamed S.H., Hamed M., Alamoudi H.A., Jastaniah Z., Alakwaa F.M. (2025). Multi-Omics Analysis of Helicobacter Pylori-Associated Gastric Cancer Identifies Hub Genes as a Novel Therapeutic Biomarker. Brief. Bioinform..

[87] Ramanathan K., Antognini D., Combes A., Paden M., Zakhary B., Ogino M., Maclaren G., Brodie D. Since January 2020 Elsevier Has Created a COVID-19 Resource Centre with Free Information in English and Mandarin on the Novel Coronavirus COVID-Research That Is Available on the COVID-19 Resource Centre-Including This for Unrestricted Research Re-Use a. 2020, 19–21. https://cmscscholar.org/wp-content/uploads/2020/06/Giovanonni-COVID-MS.pdf.

[88] Li X.-H., Li X.-H., Zhang Y.-Y., Wang M., Wang D. (2013). Atorvastatin Attenuates the Production of IL-1β, IL-6, and TNF-α in the Hippocampus of an Amyloid Β1-42-Induced Rat Model of Alzheimer’s Disease. Clin. Interv. Aging.

[89] Chan P.C., Liao M.T., Hsieh P.S. (2019). The Dualistic Effect of COX-2-Mediated Signaling in Obesity and Insulin Resistance. Int. J. Mol. Sci..

[90] Korovila I., Höhn A., Jung T., Grune T., Ott C. (2021). Reduced Liver Autophagy in High-Fat Diet Induced Liver Steatosis in New Zealand Obese Mice. Antioxidants.

[91] Masuda S., Mizukami S., Eguchi A., Ichikawa R., Nakamura M., Nakamura K., Okada R., Tanaka T., Shibutani M., Yoshida T. (2019). Immunohistochemical Expression of Autophagosome Markers LC3 and P62 in Preneoplastic Liver Foci in High Fat Diet-Fed Rats. J. Toxicol. Sci..

[92] Friuli M., Sepe C., Panza E., Travelli C., Paterniti I., Romano A. (2024). Autophagy and Inflammation an Intricate Affair in the Management of Obesity and Metabolic Disorders: Evidence for Novel Pharmacological Strategies?. Front. Pharmacol..

[93] Elsiad E.A., Abd El Aal H.A., Salem H.A., El-Yamany M.F., Rabie M.A. (2025). Liraglutide Attenuates Atorvastatin-Induced Hepatotoxicity by Restoring GLP-1R Expression and Activating Nrf2 and Autophagy Pathways in Wistar Rats. Toxics.

